# Off-Label Biologic Regimens in Psoriasis: A Systematic Review of Efficacy and Safety of Dose Escalation, Reduction, and Interrupted Biologic Therapy

**DOI:** 10.1371/journal.pone.0033486

**Published:** 2012-04-11

**Authors:** Elizabeth A. Brezinski, April W. Armstrong

**Affiliations:** 1 Department of Dermatology, University of California Davis, Sacramento, California, United States of America; 2 School of Medicine, University of California Davis, Sacramento, California, United States of America; Maastricht University Medical Center, The Netherlands

## Abstract

**Objectives:**

While off-label dosing of biologic treatments may be necessary in selected psoriasis patients, no systematic review exists to date that synthesizes the efficacy and safety of these off-label dosing regimens. The aim of this systematic review is to evaluate efficacy and safety of off-label dosing regimens (dose escalation, dose reduction, and interrupted treatment) with etanercept, adalimumab, infliximab, ustekinumab, and alefacept for psoriasis treatment.

**Data Sources and Study Selection:**

We searched OVID Medline from January 1, 1990 through August 1, 2011 for prospective clinical trials that studied biologic therapy for psoriasis treatment in adults. Individual articles were screened for studies that examined escalated, reduced, or interrupted therapy with etanercept, adalimumab, infliximab, ustekinumab, or alefacept.

**Data Synthesis:**

A total of 23 articles with 12,617 patients matched the inclusion and exclusion criteria for the systematic review. Data were examined for primary and secondary efficacy outcomes and adverse events including infections, malignancies, cardiovascular events, and anti-drug antibodies. The preponderance of data suggests that continuous treatment with anti-TNF agents and anti-IL12/23 agent was necessary for maintenance of disease control. Among non-responders, dose escalation with etanercept, adalimumab, ustekinumab, and alefacept typically resulted in greater efficacy than standard dosing. Dose reduction with etanercept and alefacept resulted in reduced efficacy. Withdrawal of the examined biologics led to an increase in disease activity; efficacy from retreatment did not result in equivalent initial response rates for most biologics. Safety data on off-label dosing regimens are limited.

**Conclusion:**

Dose escalation in non-responders generally resulted in increased efficacy in the examined biologics used to treat moderate-to-severe psoriasis. Continuous treatment with anti-TNF agents and anti-IL12/23 agent results in superior efficacy over interrupted therapy. The decision to use off-label dosing needs to account for both benefits and risks and be individualized to patients' disease severity, quality of life, and existence of comorbidities.

## Introduction

Psoriasis is a chronic, inflammatory skin disease associated with comorbidities, psychosocial impairment, and markedly reduced quality of life [1], [2]. The condition has an estimated prevalence of 2–3% of the population worldwide, including more than 4.5 million people in the US as of 2004 [3]–[5]. Psoriasis is considered an immune-mediated disorder involving T-cell activation and cytokine elaboration [6]. Recent characterization of psoriasis immuno-pathophysiology showed that cytokines, in particular tumor necrosis factor (TNF), interleukin-12 (IL-12) and interleukin-23 (IL-23) represent therapeutic targets [7]–[11]. Biologic therapies that alter these fundamentally important immunologic pathways in psoriasis have been developed [12]. Further, biologic drugs serve as welcomed alternatives to traditional systemic treatments such as methotrexate and cyclosporine that can be associated with cumulative, dose-dependent toxicities [13], [14].

### Biologic Drugs Introduction

The safety and efficacy of etanercept, adalimumab, infliximab, ustekinumab, and alefacept for the long-term treatment of adults with moderate-to-severe plaque psoriasis have been previously established in large randomized, double blind, placebo-controlled clinical trials [15]–[22]. Of particular interest are the health benefits and risks for tapering psoriasis patients off the biologic drugs etanercept, adalimumab, infliximab, ustekinumab, and alefacept. It is important to identify how long patients will stay in remission following treatment cessation and to understand the clinical characteristics associated with biologic therapy withdrawal, including the risk of disease rebound and development of anti-drug antibodies. Furthermore, it is of interest to determine whether control of psoriasis can be recaptured with retreatment following disease relapse.

### Defining Non-Standard, Off-Label Dosing Regimens

In this systematic review, “off-label” or “non-standard” dosing of biologics refers to any dosing regimens that are not the current FDA-approved regimens for psoriasis treatment. The non-standard dosing regimens are broadly categorized into (1) dose escalation or intensification, (2) dose reduction, (3) interrupted therapy followed by retreatment, and (4) intermittent therapy.

Specifically, dose escalation includes shortening the dosing interval and/or increasing the amount of medication per single dose. Similarly, dose reduction includes both lengthening of the dosing interval and/or reduction in the amount of medication per single dose. Interrupted treatment is defined as a withdrawal period followed by a retreatment period with a biologic agent; the retreatment period typically begins either at the time of disease relapse or after a predetermined period of medication interruption. In intermittent therapy, multiple treatment cycles occur punctuated by regular periods of non-retreatment.

Clinicians consider using non-standard dosing regimens to treat psoriasis patients for various reasons, including patients' unsatisfactory response to approved regimen, changing or discontinuing health insurance coverage, or preparing for surgeries with significant infectious risks. Therefore, understanding the literature on efficacy and safety of non-standard biologics dosing regimens is crucial to clinical decision-making and care for psoriasis patients.

### Aims of Systematic Review

The aims of this systematic review are (1) to determine efficacy of off-label dosing regimens of biologic treatments in adults with psoriasis, including dosing escalation, reduction, interruption with retreatment, and intermittent therapy, and (2) to assess safety of these off-label dosing regimens by examination of serious adverse events (AEs), psoriasis rebound, and anti-drug antibodies.

## Methods

### Data Sources

A systematic search was performed using Ovid MEDLINE In-Process and Ovid MEDLINE Daily from January 1, 1990 through August 1, 2011. We searched for prospective clinical trials, which included non-phase I/II randomized controlled clinical trials (RCTs) and open-label extension studies that evaluated a non-standard dosing regimen with a single biologic drug for the treatment of moderate-to-severe plaque psoriasis in adults. Initial identification of studies were performed using the search terms “psoriasis,” “etanercept,” “adalimumab,” “infliximab,” “ustekinumab,” and “alefacept.” Five searches with the term “psoriasis AND” each of the five biologic drugs were then conducted. These five searches were then combined with “OR” in a final search. Next, exclusion criteria were applied. Exclusion criteria included studies that were not in English, review articles, and pediatric evaluations. Then inclusion criteria was defined as “adults (19 plus years),” “clinical trials,” and “randomized controlled trials.” Titles and abstracts obtained from this systematic search were screened for trials that studied biologic therapy in non-standard regimens, which include dose escalation, dose reduction, interrupted therapy with retreatment, or intermittent therapy.

### Study Selection

Publications were selected based on five criteria: study design (randomized controlled clinical trials, randomized trials, and prospective open-label extension studies) study population (adults with moderate-to-severe plaque psoriasis), intervention (non-standard biologic drug dosing regimens as a single therapy), data available for the primary outcomes (PASI score, PGA score, or median time to relapse), and data available for adverse events (serious infections, malignancy, major adverse cardiovascular events [MACE], or anti-drug antibodies). Studies that combined biologic treatment with other medications, such as methotrexate, were not included in this systematic review.

### Methodological Quality of Studies

We recorded several aspects of study design including randomization, allocation concealment, groups similar at baseline, blinding (double, single, open), inclusion of all randomized participants, completeness of follow-up, and funding source.

### Data Abstraction

Data were extracted independently by two authors (EAB and AWA) and any disagreements were resolved by consensus. Studies were also graded using the Grading of Recommendations Assessment, Development and Evaluation Working Group guidelines to provide treatment recommendations based on quality of evidence and clinical outcomes [23].

## Results

### Study Selection

**Figure 1:**
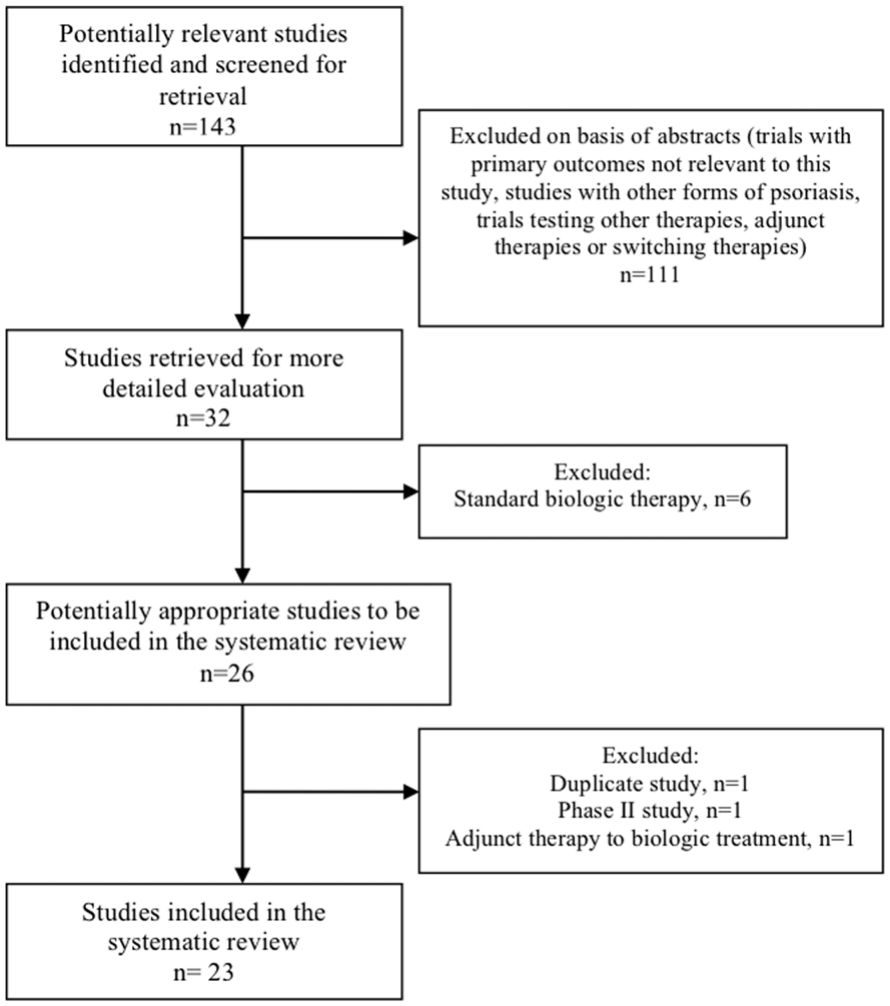
Summary of Systematic Review Study Selection.

An initial review of the databases generated 143 publications that matched the search criteria. After reading the abstracts of the relevant studies, we included 23 publications with 12,617 participants in this article (**Figure 1**). Study characteristics and primary and secondary outcomes for each drug are found in **Tables S1, S2, S3, S4, S5**. For each trial, safety data and anti-drug antibody data are recorded in **Tables S6, S7, S8, S9, S10.**

### Etanercept FDA-Approved Dosing Regimen

Etanercept is a fully human dimeric fusion protein in the class of TNF-alpha (TNF-α) inhibitors that binds both the soluble and membrane bound TNF-α and TNF-beta (TNF-β), preventing the cytokine from binding cell surface receptors [24]. The biologic drug is FDA-approved as a subcutaneous (SC) injection dosed at 50 mg twice weekly (BIW) for 3 months, followed by 50 mg once weekly (QW) for an unspecified amount of time [24]. **Table S1** summarizes the outcome data for off-label dosing regimens with etanercept from six clinical studies.

### Efficacy of Etanercept Off-Label Dosing Regimens

#### Efficacy of Etanercept Dose Escalation

From the literature review, two studies examined the efficacy of etanercept dose escalation [25], [26]. Cassano et al. studied dose escalation to 50 mg BIW etanercept in eight patients who did not achieve PASI 50 after 12 weeks of 50 mg QW etanercept [26]. At week 24, 75% (6/8) of patients dosed at 50 mg BIW etanercept achieved PASI 50 and were then discontinued from etanercept treatment. At week 36, PASI 50 was maintained by four out of these six patients [26]. Another study investigated dose escalation to 50 mg BIW after 12 weeks of maintenance dose at 50 mg QW etanercept if participants satisfied 1 of 3 criteria: (1) did not achieve PASI 75 from baseline, (2) achieved PASI 75 but had significant residual disease overall, or (3) achieved PASI 75 but had a clinically significant residual disease in a area of functional or high cosmetic importance [25]. Among the 591 patients who increased etanercept dose, the major reason was that patients did not achieve PASI 75 (83%, 492/591) [25]. Leonardi et al. compared the efficacy of 50 mg BIW dosing with standard 50 mg QW dosing. Specifically, the proportion of patients on the standard 50 mg QW dosing achieving PASI 75 were 44% at baseline, 61% at week 12, 65% at week 24, 68% at week 48, and 60% at week 72. In comparison, the proportion of patients on 50 mg BIW dose of etanercept achieving PASI 75 were 27% at baseline, 33% at week 12, 26% at week 24, 44% at week 48, and 43% at week 72 [25]. The proportion of patients on standard 50 mg QW etanercept achieving PGA of “clear” or ‘“almost clear” was 55%, 54%, and 51% at weeks 12, 48, and 72, respectively. In comparison, the proportion of those on 50 mg BIW dosing achieving “clear” or “almost clear” was 26%, 28%, and 27% at weeks 12, 48, and 72, respectively [25].

#### Efficacy of Etanercept Dose Reduction

A search of the literature identified two studies that investigated dose reduction with etanercept [16], [26]. Leonardi et al. compared 25 mg QW etanercept and 25 mg BIW etanercept to standard induction dosing of 50 mg BIW for 24 weeks [16]. At week 12, 14% of patients receiving 25 mg QW therapy and 34% of those receiving 25 mg BIW therapy achieved PASI 75 compared to 49% of patients on the standard induction dose. Similarly, at week 24, 25% of patients receiving 25 mg QW and 44% of those receiving 25 mg BIW achieved PASI 75 compared to 59% of those receiving 50 mg BIW etanercept [16]. Cassano et al. compared 24 weeks of 50 mg QW therapy to standard induction and maintenance dose etanercept [26]. At week 12, PASI 50 was achieved by 75% of patients receiving 50 mg QW induction therapy compared to 92% of patients on 50 mg BIW induction therapy (p = 0.06). Those who achieved PASI 50 at week 12 on etanercept 50 mg QW were continued on the same dose for another 12 weeks. At week 24, PASI 50 was achieved by 100% of those who were continued at 50 mg QW and 100% of those who received standard 50 mg BIW induction therapy followed by 50 mg QW maintenance etanercept [26].

#### Efficacy of Etanercept Withdrawal and Retreatment

After reviewing the literature, three studies that investigated etanercept withdrawal and retreatment were included in this article [27]–[29]. Gordon et al. studied a withdrawal period followed by 24 weeks of retreatment with a randomized dose of 50 mg BIW etanercept, 25 mg BIW etanercept, or 25 mg QW etanercept [27]. The median time to disease relapse was reported to be 85 days, which is defined as the time period during which at least half of the numerical improvement in PASI that occurred in the initial 24-week treatment and follow-up period was lost. The investigators reported that the median time to loss of PASI 75 for patients who achieved PASI 75 during the 24-week treatment cycle was 57 days [27]. Retreatment efficacy, defined as the proportion of patients who were PASI 75 responders during the initial 24 week treatment cycle that achieved PASI 75 at week 12 of the retreatment cycle, was reported to be 52% [27].

A second study by Moore et al. compared continuous versus interrupted treatment with 50 mg QW etanercept from weeks 12 to 24 [28]. Patients identified as responders (n = 917, 72.0%) were discontinued from initial etanercept therapy at week 12 and retreated with the original dose upon disease relapse. At week 24, a PGA score 0 to 2, and improvement from baseline was achieved by 71.0% of patients in the group receiving continuous therapy and 59.5% of patients who received interrupted treatment (p<0.0001), compared to 71.3% and 72.0% for each group, respectively, at week 12 [28]. The investigators also found that a PGA “clear” (0) or “almost clear” (1) status at week 12 and 24 was achieved by 48.9% and 47.2% of patients in the continuous treatment group, respectively, and 47.6% and 32.2% of patients in the interrupted treatment group, at the same time intervals [28]. The mean (median) time to relapse in the interrupted group was 36.9(33.0) days and the mean (median) time to regain responder status in patients who relapsed was 35.0(29.0) days [28].

Ortonne et al. studied etanercept retreatment in responders to therapy after a withdrawal period [29]. At week 12 of treatment with 50 mg BIW etanercept, responders to the regimen were withdrawn from treatment, and administered a reduced induction dose of 25 mg BIW etanercept upon disease relapse [29]. The mean (SD) time to relapse was 72(46) days or about 2 months. The authors found that 83% of responders recaptured a PGA score of 0 to 2 upon retreatment [29]. The mean time to response after retreatment was 15 weeks, compared to 11 weeks for the initial standard dose (p = 0.001) [29].

### Etanercept Safety Data

Safety data from the off-label etanercept regimens were examined whenever available and can be found in **Table S6**. A dose escalation study by Leonardi et al. showed a serious infections rate of 1.9 events per 100 patient-years in the 50 mg BIW compared to 0.9 events per 100 patient-years in the 50 mg QW group [25]. Leonardi et al. also reported two (2/591) myocardial infarctions in the 50 mg BIW group compared to zero (0/321) in the 50 mg QW group [25].

Safety data for withdrawal-retreatment studies were also analyzed. After a median of 85 days of etanercept withdrawal, one case (1/85) of rebound was reported in a patient receiving 25 mg etanercept QW, which was later reversed after 4 weeks of treatment re-initiation [27]. During the retreatment period, two serious infections (2/103) in patients receiving etanercept 50 mg BIW and seven unspecified serious AEs were reported [27]. Another etanercept withdrawal study reported one serious infection (1/363) during the withdrawal period [29]. During the retreatment period with 50 mg QW, Moore et al. reported three internal malignancies and nine non-melanoma skin cancers among 1,274 patients [28]. They also reported three unspecified cases of congestive heart failure and two cases of coronary artery disease in the interrupted treatment group [28].

### Etanercept Anti-Drug Antibodies

During one dose escalation study, Leonardi et al. reported non-neutralizing antibodies in 15.2% of patients, and the authors noted that antibodies developed to a similar degree in both the 50 mg BIW dose escalation group and the 50 mg QW group [25]. In a dose reduction study by Leonardi et al., the investigators found that 1.2% (8/652) of patients treated with etanercept at an unspecified dose developed antibodies [16]. After a median of 85 days of withdrawal, Gordon et al. reported that 4.7% of patients displayed low titer, non-neutralizing antibodies during the retreatment phase [27].

### Adalimumab FDA-Approved Dosing Regimen

Adalimumab is a fully human monoclonal antibody that binds specifically to TNF-α but not TNF-β. It lyses cells that express TNF-α on their surface *in vitro* [30]. Adalimumab is approved as a SC injection for the treatment of moderate-to-severe psoriasis. The FDA-approved standard dosing regimen for adalimumab is an initial dose of 80 mg at week 0 that is followed by 40 mg every other week (EOW) starting at week 1 [30]. **Table S2** presents the outcome data for off-label dosing regimens with adalimumab from three studies.

### Efficacy of Adalimumab Off-Label Dosing Regimens

#### Efficacy of Adalimumab Dose Escalation

One study investigated dose escalation therapy by administering adalimumab 40 mg QW to 30 patients who achieved <PASI 50 during the initial 24-week study period [31]. After 36 weeks of treatment (from week 24 through week 60) at 40 mg QW, 17% achieved PASI 75 and 40% achieved PASI 50 [31].

#### Efficacy of Adalimumab Withdrawal and Retreatment

Two trials examined withdrawal and retreatment with adalimumab [17], [32]. Menter et al. investigated withdrawal from treatment by re-randomizing 490 patients who had achieved PASI 75 previously to adalimumab 40 mg EOW or placebo at week 33 of the study. A patient was determined to have “lost adequate response” if he/she experienced <PASI 50 response and at least 6-point increase in PASI score relative to week 33 score. Approximately 19 weeks after randomization, 28% (68/240) of patients who were withdrawn from adalimumab lost adequate response compared with 5% (12/250) of participants who were continued to adalimumab 40 mg EOW [17].

A second study evaluated the efficacy of adalimumab for retreatment after open-label treatment and withdrawal periods [32]. Patients who achieved stable psoriasis control during the open-label treatment period were withdrawn from therapy and monitored for relapse for up to 40 weeks. Retreatment began when a patient relapsed or had achieved week 40 without relapse. Papp et al. found that the median time to relapse was 141 days (IQR 93–202 days), or almost 5 months [32]. After the withdrawal period, patients entered 16 weeks of retreatment with adalimumab 80 mg initially followed by 40 mg EOW. Among patients who had relapsed prior to week 40, 69% (123/178) achieved PGA “clear” or “minimal” by the end of the retreatment period compared to 89% (95/107) of patients who did not relapse during the withdrawal period [32]. Using PASI 75 as a secondary endpoint, the investigators found that 83% of patients who had relapsed during the withdrawal period achieved PASI 75 by the end of the retreatment period, compared to 93% of those who did not relapse [32].

### Adalimumab Safety Data

Safety data from the off-label adalimumab regimens were recorded whenever available and are can be found in **Table S7**. In the aforementioned studies, there were no reports of rebound after withdrawal of adalimumab and no cases of non-melanoma skin cancer, demyelinating disease, or lupus-like syndrome through 60 weeks of investigation [17], [31], [32].

After dose escalation to 40 mg QW adalimumab therapy, Gordon et al. reported one case (1/50) of recent-onset latent TB infection and one death (1/50) due to cerebrovascular accident in the 40 mg QW group and one case of coccidiomycosis (1/97) in the 40 mg EOW group [31]. With regard to malignancies, there were three malignancies (3/50) in the 40 mg QW group compared to two malignancies (2/97) in the 40 mg EOW group [31].

Menter et al. reported aggregate safety data for the 52-week study period and did not attribute AEs specifically to the withdrawal-retreatment phases [17]. During the 16 week retreatment period with 285 patients, Papp et al. reported one malignant melanoma in situ, two serious infections (pneumonia and hepatitis C), and seven serious AEs [32]. Specifically, the serious AEs were coronary artery disease, abdominal adhesions, umbilical hernia, chest discomfort, nephrolithiasis, and fracture of humerus [32].

### Adalimumab Anti-Drug Antibodies

No anti-adalimumab antibody (AAA) analyses were reported for adalimumab dose escalation trials. Although withdrawal-retreatment phases were included in the Menter et al. study, the authors reported aggregate AAA data for the 52-week study period and did not specify AAA in the withdrawal-retreatment phase [17]. During the 52-week period, 8.8% (73/825) of patients treated with at least one dose of adalimumab had detectable AAAs at least once [17]. A subanalysis showed that 3/7 positive AAA patients compared with 65/233 (27.9%) negative AAA patients lost adequate response during adalimumab withdrawal [17].

Papp et al. reported that after a median period of 141 days of withdrawal, 6% (17/275) of patients tested positive for AAAs [32]. During retreatment, positive AAA samples decreased to 1% (3/264) after 12 weeks and increased to 2% (4/262) at 16 weeks. A sub-analysis of patients who relapsed during the withdrawal period showed that, among those who achieved a PGA score of “clear” or “almost clear” during retreatment, 4% (5/119) had positive AAAs; in comparison, in those who failed to achieve a PGA score of “clear” or “almost clear” during retreatment, 15% (8/55) had positive AAA [32]. The authors concluded that the presence of AAAs is associated with increased risk of failure to re-achieve efficacy following treatment discontinuation and relapse [32]. In a prospective observational cohort study (n = 29), Lecluse et al. similarly observed that AAAs in psoriasis patients are associated with lower serum adalimumab trough concentrations and lack of adequate response [33]. Of note, in rheumatoid arthritis patients, the development of AAAs is associated with lower serum adalimumab concentrations, discontinued treatment, higher disease activity during treatment, and fewer instances of disease remission during 3 years of follow-up [34].

### Infliximab FDA-Approved Dosing Regimen

Infliximab is a chimeric monoclonal antibody to TNF-α that acts to neutralize soluble TNF-α and block membrane bound TNF-α [35]. This TNF-α inhibitor is administered by intravenous (IV) infusion. Infliximab is indicated for the treatment of psoriasis in a weight-based dosing regimen of 5 mg/kg at weeks 0, 2 and 6, then every 8 weeks [35]. **Table S3** presents the compiled outcome data for off-label dosing regimens with infliximab from four publications.

### Efficacy of Infliximab Off-Label Dosing Regimens

#### Efficacy of Infliximab Dose Escalation

One clinical trial specifically investigated dose intensification with infliximab in 33 patients [36]. In a controlled trial comparing an escalated dose of 10 mg/kg IV infliximab versus the standard dose of 5 mg/kg IV infliximab administered at weeks 0, 2, and 6, Chaudhari et al found that PASI 75 was achieved by 73% of patients dosed at 10 mg/kg compared to 82% of those dosed at 5 mg/kg at week 10 [36].

#### Efficacy of Infliximab Withdrawal and Retreatment

A review of the literature identified three controlled trials that investigated infliximab withdrawal and retreatment in adult psoriasis patients [37]–[39]. Gottlieb et al. conducted a 16 week open-label study at the conclusion to the aforementioned dose escalation study to investigate withdrawal and retreatment with 10 mg/kg IV infliximab and 5 mg/kg IV infliximab [39]. “Responders” to initial treatment (PGA “good,” “excellent,” or “clear”) with 10 mg/kg or 5 mg/kg IV infliximab were withdrawn and monitored for disease relapse, defined as loss of at least half of the improvement in PASI at week 10. At the time of relapse, patients were treated with a single-dose infusion of their originally randomized dose. The authors reported that patients in the 5 mg/kg group began to lose response after 8 weeks of drug withdrawal compared to 12 weeks for the 10 mg/kg group [39].

In EXPRESS II, Menter et al. evaluated continuous versus interrupted treatment with IV infliximab dosed at 3 mg/kg or 5 mg/kg [37]. All patients received infliximab 3 mg/kg or 5 mg/kg at weeks 0, 2, and 6. From week 6 through week 14, all patients entered a withdrawal period. At week 14, the patients were re-randomized to continuous infusions of infliximab at 8-week intervals, or interruption with retreatment at the originally randomized dose upon loss of PASI 75. At week 10, during the withdrawal period, 70.3% of patients on 3 mg/kg and 75.5% of patients on 5 mg/kg achieved PASI 75. At week 50, PASI 75 was achieved by 43.8% of patients on 3 mg/kg every-8-weeks, 54.5% of those on 5 mg/kg every-8-weeks, 25.4% of patients on 3 mg/kg interrupted treatment, and 38.1% of those on 5 mg/kg interrupted treatment [37]. Similar outcomes were observed using proportion of patients achieving PGA scores of “clear” (1) or “excellent” (2) at weeks 10 and 50. At week 10, 69.8% of patients receiving 3 mg/kg and 76.0% of patients receiving 5 mg/kg achieved a PGA score of 1 or 2. In comparison, at week 50, 46.9% of patients dosed at 3 mg/kg every-8-weeks, 58.2% of those at 5 mg/kg every-8-weeks, 31.7% on 3 mg/kg interrupted treatment, and 42.1% on 5 mg/kg interrupted treatment achieved a PGA score of 1 or 2 [37]. The authors reported that the most common time interval for intermittent infusions was 4 to 8 weeks [37].

Gottlieb et al. examined the effect of a single additional dose of infliximab after a 20-week withdrawal period [38]. At the study initiation, all patients were administered 3 mg/kg or 5 mg/kg IV infliximab at weeks 0, 2, and 6 [38]. From week 6 through week 26, all patients were withdrawn from infliximab treatment. During the withdrawal period at week 10, PASI 75 was achieved by 71.9% of patients on 3 mg/kg and 87.9% of patients on 5 mg/kg. At week 26, patients with PGA of “moderate” or “severe” disease (114/198) were eligible for a single additional IV infusion of the originally assigned dose of infliximab. At week 30, a PGA score of “clear,” “minimal,” or “mild” was achieved by 38% of those retreated with one single 3 mg/kg infusion and 64% of those retreated with one single 5 mg/kg infusion of infliximab [38].

### Infliximab Safety Data

Safety data from the off-label infliximab regimens were examined whenever available and can be found in **Table S8**. In the dose escalation trial, one case (1/11) of pneumonia was reported in the 10 mg/kg group and one dental abscess (1/11) was reported in the 5 mg/kg group [36]. In the withdrawal and retreatment study by Gottlieb et al, serious infections were reported in three (3/11) patients in the 10 mg/kg group, two (2/11) patients in the 5 mg/kg group, and two (2/11) patients in the placebo group [39]. During the induction phase of EXPRESS II, one case (1/313) of TB was reported in the 3 mg/kg group. During the withdrawal-retreatment phase, another case of TB was reported in the 5 mg/kg interrupted treatment group [37]. In the same study, the authors noted three cases (3/835) of lupus-like syndrome during the 50-week study period (one in placebo and two in infliximab-treated groups not further specified). Overall in the EXPRESS II study, one case of peripheral neuropathy was reported with an unspecified dose of infliximab [37]. From the aforementioned study by Gottlieb et al., one case (1/99) of sepsis was reported in a patient receiving 5 mg/kg infliximab [38].

### Infliximab Anti-Drug Antibodies

In EXPRESS II, anti-infliximab antibodies were detected in 69/145 (51.5%) patients in the 3 mg/kg every-8-week treatment group, 60/148 (46.2%) in the 3 mg/kg interrupted treatment group, 49/148 (35.8%) in the 5 mg/kg every-8-week group, and 59/149 (41.5%) in the 5 mg/kg interrupted treatment group [37]. The investigators reported that a majority (61.1%) of titers were <1∶40 [37]. Gottlieb et al. reported that 21/76 (27.6%) in the 3 mg/kg patients had antibodies to infliximab, compared to 17/87 (19.5%) in the 5 mg/kg group [38]. Infusion reactions were reported in 9/38 (24%) patients with antibodies through week 26, compared to 25/116 (22%) patients with no detectable antibodies [38].

### Ustekinumab FDA-Approved Dosing Regimen

In 2009, ustekinumab, a fully human monoclonal antibody that binds p40 subunit of IL-12 and IL-23 was approved for the treatment of moderate-to-severe psoriasis [40]. Ustekinumab is administered based on weight at 45 mg (≤100 kg) or 90 mg (>100 kg) by SC injection at weeks 0 and 4, and then every 12 weeks thereafter [40]. Outcomes for off-label dosing regimens with ustekinumab in two trials are presented in **Table S4**.

### Efficacy of Ustekinumab Off-Label Dosing Regimens

#### Efficacy of Ustekinumab Dose Escalation

In a dose escalation study, Papp et al. investigated the effect of dose intensification with ustekinumab in partial responders [21]. Specifically, patients achieving between PASI 50 and PASI 75 response at week 28 of standard dose ustekinumab (158/821) were re-randomized to continue dosing at 45 mg or 90 mg every 12 weeks or shorten the dosing interval to every 8 weeks [21]. Between weeks 40 and 52, all patients underwent four study visits. The mean number of visits that patients achieved PASI 75 was used as an outcomes measure. In patients receiving every-8-week dosing of 45 mg or 90 mg ustekinumab, the mean number of visits with PASI 75 was 1.75 visits, compared to 1.56 visits in patients dosed every 12 weeks (p = 0.468) [21]. Specifically, in the 45 mg group, the mean number of visits with patients achieving PASI 75 was 1.13 in the every-8-week dosing compared to 1.54 in the every-12-week dosing group (p = 0.21). In the 90 mg group, the mean number of visits with patients achieving PASI 75 was 2.63 in the every-8-week dosing compared to 1.58 in the every-12-week dosing group (p = 0.014) .[21].

#### Efficacy of Ustekinumab Withdrawal and Retreatment

One study investigated ustekinumab withdrawal and retreatment upon loss of responder status [20]. Leonardi et al. studied 76 weeks of treatment comparing maintenance therapy to interrupted treatment to determine time to loss of response [20]. Patients were randomly assigned to receive ustekinumab 45 mg or 90 mg at standard dosing intervals until week 40. At week 40, the patients who achieved PASI 75 were re-randomized to maintenance treatment or withdrawal of ustekinumab. Retreatment for the withdrawal group was administered upon loss of PASI 75 response. The median time to loss of response in the withdrawal group was 15 weeks or nearly 4 months [20]. Of the 195 patients who reinitiated ustekinumab after the withdrawal period, 167 (85.6%) achieved PASI 75 within 12 weeks of reinitiating therapy [20].

### Ustekinumab Safety Data

Safety data from the off-label ustekinumab regimens were examined whenever available and can be found in **Table S9**. During the dose escalation phase by Papp et al., one (1/77) cutaneous malignancy and one (1/77) non-cutaneous malignancy were reported in the every-8-week dosing group without specified dose [21]. Two (2/77) serious AEs were reported in the every-8-week dosing group without specified dose. One (1/81) serious infection and six (6/81) serious AEs were reported in the every-12-week dosing group without specified dose [21].

During the withdrawal phase of the study by Leonardi et al., one (1/160) non-cutaneous cancer was reported in the interrupted treatment group and two (2/161) cutaneous cancers were reported in the maintenance therapy group [20]. The authors also reported two (2/160) serious infections and seven (7/160) serious AEs in the interrupted treatment group and one (1/161) serious AE in the maintenance group during the randomized withdrawal phase [20].

### Ustekinumab Anti-Drug Antibodies

In the dose intensification study by Papp et al., anti-drug antibodies were found in 12.7% (20/158) of partial responders and 2.0% (12/589) of PASI 75 responders. The authors determined that most antibodies were neutralizing [21]. Overall, 5.4% (65/1202) of all patients receiving ustekinumab in the study developed antibodies [21]. In the study containing withdrawal-retreatment phases, anti-ustekinumab antibodies were found in 5.1% (38/746) of patients_ENREF_20 [20]. The majority of patients had low titers of less than 1∶320 [20].

### Alefacept FDA-Approved Dosing Regimen

Alefacept is a recombinant dimeric fusion protein that consists of the extracellular CD2-binding portion of the human leukocyte function antigen-3 (LFA-3) linked to the Fc portion of the human IgG1 [41]. In psoriasis, activation of T lymphocytes involves interaction between LFA-3 on the antigen-presenting cells and CD2 on T lymphocytes. Alefacept binds to CD2 on lymphocytes, thereby inhibiting the LFA-3/CD2 interaction. Alefacept interferes with the activation and proliferation of memory effector T lymphocytes [41]. Alefacept is approved for dosing at 15 mg by once weekly intramuscular (IM) injection for a course of 12 weeks [41]. Repeated rounds of 12-week treatment periods are considered for patients only after a 12-week drug holiday provided that CD4+ T lymphocyte counts are within the normal range [41]. We will define a retreatment cycle as 12 weeks of treatment with alefacept, followed by 12 weeks of drug-free observation. **Table S5** summarizes the outcome data for off-label dosing regimens with alefacept from seven clinical studies.

### Efficacy of Alefacept Off-Label Dosing Regimens

#### Efficacy of Alefacept Dose Escalation

Two trials that studied dose escalation regimens with alefacept were identified in a search of the literature [42], [43]. Gribetz et al. compared efficacy and safety of standard 12-week versus extended 16-week therapy with 15 mg IM alefacept in 20 patients [42]. After an initial 12 weekly dosing of alefacept, cohort 1 received 4 weekly doses of placebo (standard dosing), whereas cohort 2 received 4 additional weekly doses of alefacept (extended 16-week group). The mean percentage change in PASI from baseline during weeks 12 to 24 was 2% for cohort 1 compared to 26% for cohort 2 (p = <0.05) [42]. PASI 75 and PASI 50 were achieved by 10% and 60% of patients in cohort 1, respectively, and 30% and 60% of patients in cohort 2 at any time during weeks 12 and 24 [42]. At week 24, PGA “clear” or “almost clear” was achieved in 0/10 (0%) of those in cohort 1 and 3/10 (30%) in cohort 2 [42]. Cafardi et al. investigated treatment escalation in two off-label regimens [43]. Cohort 1 received 30 mg IM alefacept for 6 weeks followed by 6 weeks of 15 mg IM alefacept, and cohort 2 received 30 mg IM alefacept for 12 weeks. At week 14 of the study, 1/8 (12.5%) patients in cohort 1 and 1/8 (12.5%) in cohort 2 achieved PASI 75 [43].

#### Efficacy of Alefacept Dose Reduction

Ortonne et al. and Lebwohl et al. compared alefacept 15 mg IM once weekly, 10 mg IM once weekly, or placebo in 507 adult psoriasis patients [44], [45]. At week 2 of the drug holiday, after 12-weeks of treatment, PASI 75 was achieved by 21% of patients receiving 15 mg alefacept, 12% of those on 10 mg alefacept, and 5% of those receiving placebo [44].

#### Efficacy of Alefacept Intermittent Treatment

Four studies investigated intermittent treatment with alefacept defined as drug withdrawal and retreatment in consecutive cycles [22], [46]–[48]. Krueger et al. compared two cycles of 7.5 mg IV alefacept, defined as cohort 1, to one cycle of 7.5 mg IV alefacept followed by one cycle of placebo, defined as cohort 2 [22]. PASI 75 at week 2 of withdrawal during the second treatment cycle was achieved by 23% of cohort 1 compared to 7% of cohort 2 [22]. Overall response rates (ORRs) were defined as achieving PASI 75, PASI 50, or a PGA score of “clear” or “almost clear” during any time in a 24-week treatment and follow-up cycle. After one treatment cycle, members of cohorts 1 and 2 reported ORRs to be 28% (PASI 75), 56% (PASI 50), and 23% (PGA “clear” or “almost clear”) [22]. After two cycles, cohort 1 achieved higher ORRs, 37% (PASI 75), 64% (PASI 50), and 30% (PGA “clear” or “almost clear”), compared to cohort 2 who achieved ORRs of 19%, 49%, and 18% after 12 weeks of placebo (p = 0.035 for PGA score “clear” or “almost clear”) [22]. The median duration of PASI 50 maintenance in cohort 2 patients who achieved PASI 75 anytime during the initial treatment cycle was 216 days, or over 7 months [22].

Three open-label studies evaluated retreatment cycles with alefacept for up to three treatment cycles [46]–[48]. Each study enrolled patients who had previously received treatment with one or two cycles of alefacept in Phase II or III drug trials and who presented with disease severity investigators determined required systemic therapy. Lowe et al. administered 7.5 mg IV alefacept to 174 patients who had previously received alefacept in a Phase II RCT [46]. Two weeks after completion of the 12-week alefacept administration, PASI 75 was achieved by 16% of patients in cycle 1 compared to 18% of patients at the same point during treatment cycle 2 [46]. Of note, almost half (50/107) patients experienced superior ORRs during cycle 2 compared to cycle 1 [46]. Gordon et al. examined the median duration to loss of response, defined as PGA “mild” or more severe, in patients treated with 15 mg IM alefacept [48]. The authors found that patients who achieved PASI 75 during initial treatment maintained PASI 50 for a median duration of 209 days or approximately 7 months. Upon loss of response, patients were retreated with 15 mg IM alefacept for 12 weeks. At the conclusion of the 24 week study, 43% of patients had achieved PASI 75 upon retreatment with alefacept [48]. Roberts et al. investigated retreatment cycles with 15 mg IM alefacept in patients who did not receive PGA “clear” after each cycle of treatment [47]. PGA “clear” or “almost clear” was achieved by 16%, 22%, and 19% of patients at week 2 after the 12-week treatment completion for cycles 1, 2, and 3 respectively [47].

### Alefacept Safety Data

Safety data from the off-label alefacept regimens were examined whenever available and can be found in **Table S10**. In a dose escalation trial, Gribetz et al. reported two (2/10) serious infections in cohort 1, including one case of cellulitis and one case of *Helicobacter* pylori, and no (0/10) serious infections in cohort 2 [42]. In the second dose escalation study, Cafardi et al. reported two cases (2/8) of morphological change from plaque psoriasis to erythroderma in cohort 1 [43]. One patient with erythroderma was hospitalized during the 15 mg IM alefacept phase [43]. One intermittent treatment study reported five internal and cutaneous malignancies (5/174) in unspecified treatment cycles [46] and another reported 16 (16/183) internal and cutaneous malignancies in unspecified treatment cycles [47]. Specifically, Lowe et al. stated that there were no excess malignancies in alefacept-treated patients than untreated individuals [46]. Lowe et al. reported two serious infections, including one case (1/174) of pneumonia in retreatment cycle 1 and one case (1/107) of herpes zoster in retreatment cycle 2 [46]. Roberts et al. reported 15 serious infections, including one case of mycoplasmal tracheobronchitis and 14 cases of herpes simplex: four cases (4/175) in cycle 1, six cases (6/121) in cycle 2, and four cases (4/88) in cycle 3 [47].

### Alefacept Anti-Drug Antibodies

Anti-alefacept antibodies were detected in <1% to 6% of patients receiving the study drug for up to 5 retreatment cycles with no significant increase in antibodies reported with retreatment [22], [45]–[48]. One study reported decreases in anti-drug antibodies with retreatment [46]. Low titers were observed with anti-drug antibodies [22], [45]–[48] and were not associated with hypersensitivity reactions [22], [45].

## Discussion

Psoriasis is a chronic, relapsing and remitting disease that necessitates long-term treatment. In clinical practice, off-label dosing regimens are relevant and central to individualized therapy. For example, patients that exhibit sub-optimal response to standard biologic therapy may require dose intensification, whereas invasive surgery, infectious episodes, or changes in healthcare coverage may require temporary cessation of a biologic therapy. Therefore, it is important to synthesize the highest-quality available evidence for off-label regimens to inform real-world clinical practice. No guidelines exist to date for off-label use of biologic therapy in dose escalated, reduced, interrupted, or intermittent regimens. Through a systematic review, we synthesized data from 23 trials that investigated non-standard treatments with etanercept, adalimumab, infliximab, ustekinumab, and alefacept for moderate-to-severe plaque psoriasis in adults.

### Dose Escalation Considerations

Most dose escalation trials were performed in patients who did not exhibit full response to standard biologic dosing regimens. For etanercept dose escalation, data support that dose escalation to 50 mg BIW among non-responders improves PASI 50 response after 12 weeks of dose intensification [26] and improves PASI 75 response for at least 60 weeks of treatment [25]. With regard to adalimumab dose escalation, in non-responders to standard therapy, dose escalation to 40 mg QW improves clinical response from 0% achieving PASI 50 to 40% achieving PASI 50 after 36 weeks of treatment [31]. Dose escalation with infliximab at 10 mg/kg did not produce superior results to standard therapy, with 73% of patients on 10 mg/kg achieving PASI 75 compared to 82% of patients on 5 mg/kg [36]. Data from one ustekinumab dose escalation study suggests that partial responders who escalate dosing to every 8 weeks experience greater psoriasis control for at least 28 weeks [21]. With regard to alefacept dose escalation, Gribetz et al. reported that an additional four weeks of alefacept resulted in greater efficacy as measured by PASI 75 and PGA than the standard 12-week dosing [42]. Larger studies are necessary to further characterize the degree of efficacy gained through the varying dose escalation regimens and duration of dose escalation.

### Dose Reduction Considerations

All dose reduction studies found that decreased doses of biologic therapy resulted in worse outcomes compared to standard biologic treatment [16], [44], [45]. Reduction of etanercept induction and maintenance treatment to 25 mg QW and 25 mg BIW resulted in decreased PASI improvement compared to standard dosing [16]. Alefacept dose reduction from 15 mg IM to 10 mg IM resulted in smaller proportion of patients achieving PASI 75 [44], [45]. Overall, treatment with FDA-approved dosing regimens resulted in superior efficacy compared to dose reduction regimens.

### Withdrawal and Retreatment Considerations

The preponderance of data in **Tables S1, S2, S3, S4, S5** suggests that continuous therapy is recommended for all biologics reviewed here with the exception of alefacept. In most studies, only responders (usually defined as patients achieving PASI 75 or PGA “clear” or “almost clear”) were eligible to enter the withdrawal and retreatment periods. Thus, much of the withdrawal-retreatment data cannot be directly extrapolated to non-responders. Studies also used different definitions for disease relapse as well as efficacy outcome measures for retreatment. Specifically, for patients on etanercept, it took a median of 85 days for responders to relapse (loss of PASI 75), and 52% of the initial responders regained PASI 75 with retreatment [27]. For those on adalimumab, nearly 5 months of treatment withdrawal was necessary for responders to relapse to at least moderate disease, and 87% of patients regained PASI 75 after retreatment [32]. Due to the significant concern with anti-drug antibody formation, continuous infliximab therapy was necessary to maintain psoriasis control. While some clinicians add methotrexate to infliximab to prevent the formation of anti-drug antibody and to increase efficacy, large RCTs are necessary to determine potential additive efficacy from methotrexate addition. Patients on ustekinumab lost PASI 50 response after a median of 15 weeks of withdrawal, and 85.6% of initial responders regained PASI 75 [20]. Alefacept is approved as intermittent treatment where treatment cycles lasting 12-weeks are punctuated with a 12-weeks of drug-free period [22], [46]–[48].

### Safety Considerations

Safety considerations are important in assessing benefit-risk profile of administering biologic therapy to eligible psoriasis patients. Challenges in assessing safety issues include low event rates, lack of a comparison group for some open-label extension studies, inconsistent reporting methods, and long lead-time expected for certain types of adverse events (such as malignancy). For standard dosing regimen of anti-TNF agents, a meta-analysis studying the association of short-term use of anti-TNF agents with infections and malignancies showed that there was a small risk of overall infection and no increased risk of serious infection or malignancy [49]. A recent meta-analysis studying adverse cardiovascular events found no significant differences in the MACE in psoriasis patients receiving etanercept, adalimumab, infliximab, or ustekinumab [50].

Most studies involving off-label dosing regimens of biologic agents enrolled a smaller number of participants, and therefore adverse event rates were small and difficult to interpret. Furthermore, the aforementioned challenges in reporting safety events likely exist to a greater degree in off-label dosing studies. While this systematic review synthesized detailed rates of adverse events for the off-label dosing regimen, further large studies are necessary to understand whether safety issues exist with dose intensification, reduction, or withdrawal-retreatment.

### Summary

Off-label dosing of biologics for the treatment of moderate-to-severe psoriasis is a clinically relevant and important issue in real-world practice settings. This is the first systematic review to date that examined off-label dosing regimens of the FDA-approved biologic agents etanercept, adalimumab, infliximab, ustekinumab, and alefacept for moderate-to-severe psoriasis. In general, the preponderance of data suggests that continuous treatment with anti-TNF agents and anti-IL12/23 agent are necessary for maintenance of disease control. Among non-responders, dose escalation with etanercept, adalimumab, and ustekinumab usually results in greater efficacy than standard dosing. Safety data on off-label dosing regimens are limited in the examined biologics, and larger studies are necessary to determine risks associated with varying dosing regimens.

In patients where interrupted therapy is considered, such as those experiencing active infections, undergoing invasive surgeries, or desiring to discontinue treatment in pregnancy, the clinician needs to carefully weigh the benefit-risk ratio of interrupted therapy. Clinicians need to consider whether an interruption is necessary, how the duration of interruption may affect subsequent treatment efficacy, and possible disease exacerbation during interruption. Therefore, the decision to use off-label dosing needs to account for both benefits and risks and be individualized to patients' disease severity, quality of life, and existence of comorbidities.

## Supporting Information

Table S1 **Etanercept Off-label Regimens: Study Characteristics and Outcomes.**
(DOCX)

Table S2 **Adalimumab Off-label Regimens: Study Characteristics and Outcomes.**
(DOCX)

Table S3 **Infliximab Off-label Regimens: Study Characteristics and Outcomes.**
(DOCX)

Table S4 **Ustekinumab Off-label Regimens: Study Characteristics and Outcomes.**
(DOCX)

Table S5 **Alefacept Off-label Regimens: Study Characteristics and Outcomes.**
(DOCX)

Table S6 **Safety Data for Etanercept Off-Label Regimens.**
(DOCX)

Table S7 **Safety Data for Adalimumab Off-Label Regimens.**
(DOCX)

Table S8 **Safety Data for Infliximab Off-Label Regimens.**
(DOCX)

Table S9 **Safety Data for Ustekinumab Off-Label Regimens.**
(DOCX)

Table S10 **Safety Data for Alefacept Off-Label Regimens.**
(DOCX)
