# Supplementary material for: Off-Label Biologic Regimens in Psoriasis: A Systematic Review of Efficacy and Safety of Dose Escalation, Reduction, and Interrupted Biologic Therapy
Source: PLoS One. 2012 Apr 11;7(4):e33486. doi: 10.1371/journal.pone.0033486 (PMC3324468; doi:10.1371/journal.pone.0033486)
Supplement: Table S1 — Etanercept Off-label Regimens: Study Characteristics and Outcomes. (DOCX) [file pone.0033486.s001.docx]

| **Table S1. Etanercept: Efficacy of Off-Label Regimens** | | | | | | | |
| --- | --- | --- | --- | --- | --- | --- | --- |
| **Dose Escalation or Reduction** | | | | | | | |
| Author, Year (Location) Study Design | N | Age mean (SD) | Gender n(%) male | Dose Escalation or Reduction | Duration of Follow-up | Primary Outcome | Secondary Outcome |
| Leonardi et al., 2003 (US), RCT Phase III [[1](#_ENREF_1)] | 652 | 25mg QW^††^: 44.4(0.9)  25mg BIW^¶^: 45.4(1.0)  50mg BIW: 44.8(0.8)  Placebo: 45.6(1.0) | 25mg QW: NR^†^ (74%)  25mg BIW: NR(67%)  50mg BIW: NR(65%)  Placebo: NR(63%) | Dose Reduction for 24 weeks: 25mg QW (n=160)  Maintenance Dose for 24 weeks: 25mg BIW (n=162)  Induction dose for 24 weeks: 50mg BIW (n=164) | Through 24 weeks | **PASI 75 at week 12**  25mg QW: 23/160 (14%)  25mg BIW: 55/162 (34%)  50mg BIW: 81/164 (49%)  Placebo: 6/166 (4%)  (p<0.001 for comparison of all doses to placebo)  **PASI 75 at week 24**  25mg QW: 40/160 (25%)  25mg BIW: 71/162 (44%)  50mg BIW: 97/164 (59%) | **PASI 50 at week 12**  25mg QW: 65/160 (41%)  25mg BIW: 94/162 (58%)  50mg BIW: 121/164 (74%)  Placebo: 24/166 (14%)  (p<0.001 for comparison of all doses to placebo)  **PASI 90 at week 12**  25mg QW: 5/160 (3%)  25mg BIW: 19/162 (12%)  50mg BIW: 36/164 (22%)  Placebo: 1/166 (1%)  **PGA “clear” or “almost clear” at week 12**  25mg QW: 37/160 (23%)  25mg BIW: 55/162 (34%)  50mg BIW: 81/164 (49%)  Placebo: 8/166 (5%)  (p<0.001 for comparison of all doses to placebo)  **PGA “clear” or “almost clear” at week 24**  25mg QW: 41/160 (26%)  25mg BIW: 63/162 (39%)  50mg BIW: 90/164 (55%) |
| Cassano et al., 2010 (Europe), Open-label [[2](#_ENREF_2)] | 72 | 50mg QW: 51.2(NR)  50mg BIW: 44.5(NR) | 50mg QW: 28(78%)  50mg BIW: 28(78%) | Phase 1: weeks 0-12  Dose Reduction: 50mg QW for 12 weeks (n=36)  Standard Dose 50mg BIW for 12 weeks (n=36)  Phase 2: weeks 12-36  Dose Reduction (QW/QW): Pts originally on 50mg QW who achieved PASI 50 (“responders”) were continued on 50mg QW to week 36 (n=27)  Dose Escalation (QW/BIW): Pts originally on 50mg QW who did not achieve PASI 50 (“non-responders”) underwent dose escalation to 50mg BIW to week 24 (n=8)  Standard Dose (BIW/QW): Pts originally on 50mg BIW who achieved PASI 50 (“responders”) were continued on 50mg QW etanercept to week 24 (n=33) | At week 24, pts in the standard dose group (BIW/QW, n=33) and the dose escalation group (QW/BIW, n=6) who maintained PASI 50 were withdrawn from therapy and evaluated at week 36. Pts in the dose reduction group (QW/QW) were continued on 50mg QW therapy until week 36. | **PASI 50 at week 12**  50mg QW: 75% (27/36)  50mg BIW: 92% (33/36)  (p=0.06)  **PASI 50 at week 24**  50mg QW/QW “responders”: 100% (27/27)  50mg QW/BIW “non-responders”: 75% (6/8)  50mg BIW/QW: 100% (33/33)  **PASI 50 at week 36**  50mg QW/QW “responders”: 93% (25/27)  50mg QW/BIW “non-responders”: 67% (4/6)  50mg BIW/QW: 70% (23/33)  (p=0.027 for QW/QW vs. BIW/QW) | **Disease relapse, defined as loss of PASI 50, at week 36 in standard dose group (BIW/QW)**  30% (10/33) of pts after an average of 6 weeks  **PASI 75 at week 12**  50mg QW: 36% (13/36)  50mg BIW: 53% (19/36)  (p=0.23)  **PASI 75 at week 24**  50mg QW/QW “responders”: 70% (19/27)  50mg QW/BIW “non-responders”: 38% (3/8)  50mg BIW/QW: 88% (29/33)  **PASI 75 at week 36**  50mg QW/QW “responders”: 63% (17/27)  50mg QW/BIW “non-responders”: 50% (3/6)  50mg BIW/QW: 64% (21/33) |
| Leonardi et al., 2010, (Canada, Europe, US) Open-label [[3](#_ENREF_3)] | 912 | 45.9(11.9) | 617(67.7%) | Dose Escalation:  At week 12, pts considered “non-responders” escalate to 50mg BIW. “Non-response” is defined as fulfillment of 1 of the following criteria (n=591):  1) Did not achieve PASI 75 from baseline of parent study (n=492/591 [83%])  2) Achieved PASI 75 but had significant residual disease (88/591 [14%])  3) Achieved PASI 75 but had clinically significant residual disease in an area of high cosmetic or functional importance (16/591 [3%]) | Through 72 weeks | NA^‡^ | **PASI 50 and PASI 75: baseline, 12, 24, 48, 72 weeks**  PASI 50  “Non-responder” group 50mg QW/BIW: 62%, 70%, 66%, 82%, 83%  (“Responder” group at 50mg QW: 76%, 90%, 90%, 91%, 90%)  PASI 75  “Non-responder” group 50mg QW/BIW: 27%, 33%, 26%, 44%, 43%  (“Responder” group at 50mg QW: 44%, 61%, 65%, 68%, 60%)  **Mean percentage improvement in PASI score from baseline, 12, 48, 72 weeks**  “Non-responder” group 50mg QW/BIW: 53.5%, 60.4%, 67.3%, 67.1%  (“Responder” group at 50mg QW: 64.8%, 75.4%, 77.4%, 75.8%)  **PGA of “clear” or “almost clear” at weeks 12, 48, 72**  “Non-responder” group 50mg QW/BIW: 26%, 28%, 27%  (“Responder” group at 50mg QW: 55%, 54%, 51%) |
| **Withdrawal & Retreatment** | | | | | | | |
| Author, Year (Location) Study Design | N | Age mean (SD) | Gender n(%) male | Withdrawal Period | Retreatment Period | Primary Outcome | Secondary Outcome |
| Gordon et al., 2006 (US), RCT [[4](#_ENREF_4)] | 652 | Overall: 45.1(12.2)  50mg BIW: 45.6(10.1)  25mg BIW: 44.7(13.0)  25mg QW: 44.4(11.6)  Placebo/25 BIW: 45.8(12.6) | Overall: NR(67%)  50mg BIW: NR(65%)  25mg BIW: NR(71%)  25mg QW: NR(74%)  Placebo/25mg BIW: NR(62%) | Pts who achieved PASI 50 at week 24 of initial treatment (n=409) discontinued etanercept until disease relapse (loss of >50% week 24 PASI improvement).  PASI 50 patients by dose: n=95/166 placebo crossover to 25mg BIW; n=85/160 25mg QW; n=107/162 25mg BIW; n=122/164 50mg BIW | Pts were retreated for 24 weeks with etanercept at originally randomized dose (25mg BIW, 25mg QW, 25mg BIW) | **Time to Disease Relapse** (=time to loss of PASI 50 response after the 24 week treatment period)  Median time to relapse: 85 days  **Measure of Retreatment Efficacy** (=numerical differences in PASI scores between week 12 of initial treatment and week 12 of retreatment)  Week 12 of initial treatment: mean PASI 5.8  Week 12 of retreatment: mean PASI 6.4  Mean difference per patient: -0.05, (95% CI -1.1 to 0) | **For the subset of pts who achieved PASI 75 at week 24 (n=252):**  **(1) Median Time to loss of PASI 50**: 91 days  (lower 25% of pts 58 days, upper 25% of pts 170 days)  **(2) Median Time to loss of PASI 75**: 57 days  **Retreatment Period Efficacy (n=297): Proportions of PASI 50 and PASI 75 responders at week 12 of initial treatment who achieved PASI 50 or 75 at week 12 of retreatment**  PASI 50 to PASI 50: Overall: 83%  50mg BIW: 87%  25mg BIW: 89%  25mg QW: 71%  Placebo/25mg BIW: 81%  PASI 75 to PASI 50: Overall: 93%  PASI 75 to PASI 75: Overall: 52%  50mg BIW: 60%  25mg BIW: 56%  25mg QW: 14%  Placebo/25 mg BIW: 53% |
| Moore et al., 2007 (US), EASE RCT & open-label [[5](#_ENREF_5)] | 2546 | Overall: 45.4(NR)  Continuous: 45.8(13.6)  Interrupted: 44.9(13.6) | Overall: NR(62%)  Continuous: NR(63%)  Interrupted: NR(62%) | Continuous 50mg QW (n=1272) treatment or interrupted (n=1274) treatment during weeks 12 to 24  Interrupted treatment: “responders” to treatment at week 12 (PGA≤2 and improvement from baseline) discontinued drug until relapse at week 16 or 20 (loss of PGA≤2) | At the time of relapse, patients were retreated with 50mg QW up to week 24 of the study | **Proportion of responders (PGA≤2 and improvement from baseline) at week 12, 24**  Continuous: 71.3%, 71.0%  Interrupted: 72.0%, 59.5%  (p<0.0001 for comparison at week 24) | **PGA “clear” or “almost clear” at weeks 12, 24**  Continuous: 48.9%, 47.2%  Interrupted: 47.6%, 32.2%  **Mean (median) time to:**  Relapse: 39.6(33.0) days  Regain responder status: 35.0(29.0) days |
| Ortonne et al., 2009 (NR), CRYSTEL RCT & open-label *post hoc analysis* [[6](#_ENREF_6)] | 226 post-hoc | Total: 44.7(12.0)  Responders: 44.9(12.1) | Total: NR(69%)  Responders: NR(68%) | At week 12, “responders” (PGA≤2) to 50mg BIW etanercept (n=226) were withdrawn from treatment | Upon relapse of PGA>3, pts were retreated with 25mg BIW etanercept upon relapse up to week 54 of the study | **Recapture PGA≤2 after retreatment**  187/226 (83%) | **Mean (SD), Median time of withdrawal**: 72(46), 51 days  **Mean time to response (PGA≤2) to etanercept**  Initial treatment: 11 weeks  Retreatment: 15 weeks  (p=0.001) |

QW ^††^ = Once weekly

BIW ^¶^ = Twice weekly

NR ^†^ = Not reported

NA ^‡^ = Not applicable to the aims of this study
