# Supplementary material for: Off-Label Biologic Regimens in Psoriasis: A Systematic Review of Efficacy and Safety of Dose Escalation, Reduction, and Interrupted Biologic Therapy
Source: PLoS One. 2012 Apr 11;7(4):e33486. doi: 10.1371/journal.pone.0033486 (PMC3324468; doi:10.1371/journal.pone.0033486)
Supplement: Table S2 — Adalimumab Off-label Regimens: Study Characteristics and Outcomes. (DOCX) [file pone.0033486.s002.docx]

| **Table S2. Adalimumab: Efficacy of Off-Label Regimens** | | | | | | | |
| --- | --- | --- | --- | --- | --- | --- | --- |
| **Dose Escalation** | | | | | | | |
| Author, Year (Location) Study Design | N | Age mean(SD) | Gender n(%) male | Dose Escalation | Duration of Follow-up | Primary Outcome | Secondary Outcome |
| Gordon et al., 2006 (Canada, US) RCT [[7](#_ENREF_7)] | 147 | 40mg QW^††^: 44(NR^†^)  40mg eow^§^: 46(NR)  Placebo: 43(NR) | 40mg QW: NR(66%)  40mg eow: NR(71%)  Placebo: NR(65%) | Dose Escalation:  Open label weeks 25-60: At week 24, if pts were “non-responders” with < PASI 50 to standard adalimumab therapy (placebo/40mg eow [n=18] and 40mg eow [n=12]) they were eligible to increase dosing to 40mg QW | Open-label Period: “Non-responders” received 40mg QW from weeks 25 to 60 | **PASI 50 at week 60**  40mg QW: 40%  **PASI 75 at week 60**  40mg QW: 17% | NR |
| **Withdrawal & Retreatment** | | | | | | | |
| Author, Year (Location) Study Design | N | Age mean(SD) | Gender n(%) male | Withdrawal Period | Retreatment Period | Primary Outcome | Secondary Outcome |
| Menter et al., 2008 (Canada, US), RCT Phase III [[8](#_ENREF_8)] | 1212 | 40mg eow:  44.1(13.2)  Placebo:  45.4(13.4) | 40mg eow: 546(67.1%)  Placebo:  257(64.6%) | Pts who achieved ≥PASI 75 at week 16 and at week 33 of the study were re-randomized to withdrawal from treatment (n=240) or to continuous treatment with 40mg eow (n=250) | NA^‡^ | **Percentage of pts “losing an adequate response” (<PASI 50 response and a 6-point increase in PASI score relative to week 33 score) after week 33 and on or before week 52**  Withdrawal from treatment: 28% (68/240)  Continuous treatment with 40mg eow: 5% (12/250)  (p<0.001) | NA |
| Papp et al., 2011 (Canada, Europe, US), Open-label [[9](#_ENREF_9)] | 1468 | mITT*: 46.0(12.69)  *mITT: Subgroup of interest that had stable psoriasis control on 40mg eow dosing defined as PGA 0 or 1 at the last two study visits of the initial open—label period | Withdrawal Period: mITT-W  73.8%  Retreatment Period: mITT-R 74.7% | Pts achieving PGA ≤2 were withdrawn from therapy after open-label period until relapse or 40 weeks (mITT-W = 347) | Pts were retreated for 16 weeks upon relapse (PGA≥3) in the withdrawal period (n=178) or when they reached a maximum of 40 weeks of withdrawal (n=107) with 80mg at week 0 followed by 40mg eow staring at week 1 to week 15 (mITT-R = 285) | **PGA “clear” (0) or “minimal” (1) at week 16 of retreatment**  Overall: 76%  For pts who relapsed during the withdrawal period: 69% (123/178)  For pts who did not relapse in withdrawal period: 89% (95/107) | **Median time to relapse for mITT-W population**  141 days (interquartile range 93-202 days)  For those that did not relapse but entered retreatment period, the median time between the end of open-label treatment and retreatment was 141 days  **PASI 75/90/100 for mITT-R at week 16 of the retreatment period**  87%/64%/34%  PASI 75 for pts in mITT-R group who relapsed was 83% compared with 93% for those who did not relapse |

QW ^††^ = Once weekly

eow ^§^ = Every other week

NR ^†^ = Not reported

NA ^‡^ = Not applicable to the aims of this study
