# Supplementary material for: Off-Label Biologic Regimens in Psoriasis: A Systematic Review of Efficacy and Safety of Dose Escalation, Reduction, and Interrupted Biologic Therapy
Source: PLoS One. 2012 Apr 11;7(4):e33486. doi: 10.1371/journal.pone.0033486 (PMC3324468; doi:10.1371/journal.pone.0033486)
Supplement: Table S3 — Infliximab Off-label Regimens: Study Characteristics and Outcomes. (DOCX) [file pone.0033486.s003.docx]

| **Table S3. Infliximab: Efficacy of Off-Label Regimens** | | | | | | | |
| --- | --- | --- | --- | --- | --- | --- | --- |
| **Dose Escalation** | | | | | | | |
| Author, Year (Location) Study Design | N | Age mean (SD) | Gender n(%) male | Dose Escalation | Duration of Follow-up | Primary Outcome | Secondary Outcome |
| Chaudhari et al., 2001 (US), RCT [[10](#_ENREF_10)] | 33 | 10mg/kg: 35(11)  5mg/kg: 51(14)  Placebo: 45(12) | 10mg/kg: 8(73%)  5mg/kg: 7(64%)  Placebo: 8(73%) | Dose Escalation: IV 10mg/kg infliximab at weeks 0, 2, and 6 (n=11)  Standard Dose: IV 5mg/kg infliximab at weeks 0, 2, and 6 (n=11) | 10 weeks | **PGA “good,” “excellent,” or “clear” at week 10**  10mg/kg: 10/11 (91%)  5mg/kg: 9/11 (82%)  Placebo: 2/11 (18%)  (p=0.0019 for 10mg/kg vs. placebo and p=0.0089 for 5mg/kg vs. placebo)  **PGA “excellent” or “clear” at week 10**  10mg/kg: 7/11 (64%)  5mg/kg: 9/11 (82%)  Placebo: 2/11 (18%) | **PASI 75 at week 10**  10mg/kg: 8/11 (73%)  5mg/kg: 9/11 (82%)  Placebo: 2/11 (18%)  (p=0.03 for 10mg/kg vs. placebo and p=0.0089 for 5mg/kg vs. placebo) |
| **Withdrawal & Retreatment** | | | | | | | |
| Author, Year (Location) Study Design | N | Age mean (SD) | Gender n(%) male | Withdrawal Period | Retreatment Period | Primary Outcome | Secondary Outcome |
| Gottlieb et al., 2003 (US), Open-label [[11](#_ENREF_11)] | 33 | 10mg/kg: 35(11)  5mg/kg: 51(14)  Placebo: 45(12) | 10mg/kg: 8(73%)  5mg/kg: 7(64%)  Placebo: 8(73%) | After IV 10mg/kg infliximab (n=11) or 5mg/kg infliximab (n=11) at weeks 0, 2, and 6, “responders” (PGA “good” “excellent” or “clear”) were withdrawn until disease relapse. | At time of relapse (loss of at least half improvement in PASI score achieved at week 10), pts (n=9) were treated with a single-dose infusion of their originally randomized dose of infliximab through week 26.  10mg/kg: 3/9 received one infusion  5mg/kg: 3/9 received one infusion, 3/9 received two infusions | **Maintenance of PASI 75, PASI 50 through week 26 (retreatment not specified)**  10mg/kg: 67%, 73%  5mg/kg: 33%, 40% | **Time to loss of response**  5mg/kg: after week 14  10mg/kg: after week 18  **Time to first retreatment**  Ranged from week 22 to week 34 |
| Gottlieb et al., 2004 (US), RCT [[12](#_ENREF_12)] | 249 | Overall: 44(NR^†^)  3mg/kg: 45(NR)  5mg/kg: 44(NR) | Overall: 174(70%)  3mg/kg: 70(NR)  5mg/kg: 73(NR) | Pts who received 3mg/kg (n=99) or 5mg/kg (n=99) IV infliximab at weeks 0, 2, and 6 were withdrawn from therapy from weeks 6 to 26 | At week 26, pts with PGA “moderate” or “severe” were eligible for a single IV dose of their originally randomized assigned treatment (n=114) | **PASI 75 at week 10**  3mg/kg: 71/99 (71.9%)  5mg/kg: 87/99 (87.9%)  Placebo: 3/51 (5.9%)  (p<0.0001 for each comparison vs. placebo) | **PGA “clear” or “minimal” at week 10**  3mg/kg: 71/99 (71.7%)  5mg/kg: 89/99 (89.9%)  Placebo: 5/51 (9.8%)  (p<0.0001 for each comparison vs. placebo)  **PGA clear, minimal, or mild at week 30 after retreatment at week 26**  3mg/kg: 38%  5mg/kg: 64%  Placebo: 18% |
| Menter et al., 2007 (Canada, Europe, US), EXPRESS II RCT [[13](#_ENREF_13)] | 835 | 3mg/kg: 43.4(12.6)  5mg/kg: 44.5(13.0) | 3mg/kg: NR(65.8%)  5mg/kg: NR(65.0%) | Pts who received 3mg/kg (n=313) or 5mg/kg (n=314) IV infliximab at weeks 0, 2, and 6 were withdrawn from therapy from weeks 6 to 14 | At week 14, pts were re-randomized to continuous infusions at 8-week intervals or intermittent (infusion when loss of PASI 75) regimens at their induction dose (n=595) | **PASI 75 at week 10 (week 4 of withdrawal period)**  3mg/kg: 70.3%  5mg/kg: 75.5%  Placebo: 1.9%  (p<0.001)  **Intermittent infusion interval**  Most common time interval for intermittent infusions was 4-8 weeks (data not shown) | **PGA “clear” or “excellent” at week 10 (week 4 of withdrawal period)**  3mg/kg: 69.8%  5mg/kg: 76.0%  Placebo: 1%  (p<0.001)  **PGA “clear” or “excellent” at week 50**  3mg/kg every-8-week: 46.9%  3mg/kg interrupted: 31.7%  5mg/kg every-8-week: 58.2%  5mg/kg interrupted: 42.1%  **PASI 90 at week 10 (week 4 of withdrawal period)**  3mg/kg: 37.1%  5mg/kg: 45.2%  Placebo: 0.5%  **PASI 75 at week 50**  3mg/kg every-8-week: 43.8%  3mg/kg interrupted: 25.4%  5mg/kg every-8-week: 54.5%  5mg/kg interrupted: 38.1% |

NR ^†^ = Not reported
