# Supplementary material for: Off-Label Biologic Regimens in Psoriasis: A Systematic Review of Efficacy and Safety of Dose Escalation, Reduction, and Interrupted Biologic Therapy
Source: PLoS One. 2012 Apr 11;7(4):e33486. doi: 10.1371/journal.pone.0033486 (PMC3324468; doi:10.1371/journal.pone.0033486)
Supplement: Table S4 — Ustekinumab Off-label Regimens: Study Characteristics and Outcomes. (DOCX) [file pone.0033486.s004.docx]

| **Table S4. Ustekinumab: Efficacy of Off-Label Regimens** | | | | | | | |
| --- | --- | --- | --- | --- | --- | --- | --- |
| **Dose Escalation** | | | | | | | |
| Author, Year (Location) Study Design | N | Age mean (SD) | Gender n(%) male | Dose Escalation | Duration of Follow-up | Primary Outcome | Secondary Outcome |
| Papp et al., 2008 (Europe, North America), RCT Phase III [[14](#_ENREF_14)] | 1230 | 45 mg: 45.1(12.1)  90 mg: 46.6(12.1)  Placebo: 47.0(12.5) | 45 mg: 283(69.2%)  90mg: 274(66.7%)  Placebo: 283(69.0%) | At week 28, partial responders (pts achieving PASI 50 to 75) to 45mg [n=93] or 90mg [n=65] ustekinumab were re-randomized to:  Continue dosing every 12 weeks: 45 mg/12weeks (n=48) or 90mg/12weeks (n=33)  Escalate to dosing every 8 weeks: 45mg/8weeks (n=45) or 90mg/8weeks (n=32) | Through week 56 | NA^‡^ | **Mean number of visits with PASI 75 response between weeks 40 and 52 in group receiving drugs every 8 weeks compared to drug every 12 weeks (total of four visits at unspecified time intervals)**  8 week group: 1.75  12 week group: 1.56  (p=0.468)  45 mg/8weeks: 1.13  45mg/12weeks: 1.54  (p=0.210)  90mg/8weeks: 2.63  90mg/12weeks: 1.58  (p=0.014) |
| **Withdrawal & Retreatment** | | | | | | | |
| Author, Year (Location) Study Design | N | Age mean (SD) | Gender n(%) male | Withdrawal Period | Retreatment Period | Primary Outcome | Secondary Outcome |
| Leonardi et al., 2008 (Belgium, Canada, US), RCT Phase III [[15](#_ENREF_15)] | 766 | 45 mg: 44.8(12.5)  90 mg: 46.2(11.3)  Placebo: 44.8 (11.3) | 45mg: 175(68.6%)  90mg: 173(67.6%)  Placebo: 183(71.8%) | At week 40, pts achieving PASI 75 at weeks 28 and 40 were re-randomized to:  Maintenance drug: 45mg (n= 77) or 90mg (n=85)  Withdrawal from treatment until loss of response: 45mg (n=73) or 90mg (n=87) | Pts were retreated at their originally assigned dose of 45mg or 90mg ustekinumab upon disease relapse, defined as loss of at least 50% PASI improvement from week 40, through week 76 (n=195) | NR^†^ | **Time to PASI 75 response in pts with maintenance ustekinumab compared with pts withdrawn from treatment at week 40**  Maintenance of PASI 75 was better in pts receiving maintenance therapy than in pts withdrawn from therapy through at least 1 year (p<0.0001)  Maintenance group: median percentage improvement in PASI remained stable to at least week 76  Withdrawal group: median percentage improvement in PASI remained stable to week 44  **Median time to loss of PASI 75 during withdrawal period** About 15 weeks  **PASI 75 within 12 weeks of retreatment**  167/195 (85.6%) |

NA ^‡^ = Not applicable to the aims of this study

NR ^†^ = Not reported
