# Supplementary material for: Off-Label Biologic Regimens in Psoriasis: A Systematic Review of Efficacy and Safety of Dose Escalation, Reduction, and Interrupted Biologic Therapy
Source: PLoS One. 2012 Apr 11;7(4):e33486. doi: 10.1371/journal.pone.0033486 (PMC3324468; doi:10.1371/journal.pone.0033486)
Supplement: Table S5 — Alefacept Off-label Regimens: Study Characteristics and Outcomes. (DOCX) [file pone.0033486.s005.docx]

| **Table S5. Alefacept: Efficacy of Off-Label Regimens** | | | | | | | |
| --- | --- | --- | --- | --- | --- | --- | --- |
| **Dose Escalation or Reduction** | | | | | | | |
| Author, Year (Location) Study Design | N | Age mean (SD) | Gender n(%) male | Dose Escalation or Reduction | Duration of Follow-up | Primary Outcome | Secondary Outcome |
| Gribetz et al., 2005 (US), RCT & open-label [[16](#_ENREF_16)] | 20 | 50(NR^†^) | NR(65) | IM alefacept 15mg QW^††^ for 12 weeks followed by:  Standard treatment: Cohort 1 (n=10) received 4 weeks IM placebo QW  Dose escalation: Cohort 2 (n=10) received 4 weeks IM alefacept 15mg QW | Through 24 weeks | **Mean percentage change in PASI from**:  Baseline PASI to week 24:  Cohort 1: 40%  Cohort 2: 62%  (p=0.10)  Week 12 to week 20:  Cohort 1: 14%  Cohort 2: 39%  (p<0.05)  Week 12 to 24:  Cohort 1: 2%  Cohort 2: 36%  (p<0.05) | ***Overall Response Rate:**  **1) PASI 75**  **2) PASI 50**  **3) PGA “clear” or “almost clear”**  ***achieved at any time during weeks 12-24**  Cohort 1:  1) 1/10(10%)  2) 6/10(60%)  3) 0/10(0%)  Cohort 2:  1) 3/10(30%)  2) 6/10(60%)  3) 3/10(30%) |
| Lebwohl et al., 2003 [[17](#_ENREF_17)] and Ortonne et al., 2003 [[18](#_ENREF_18)] (Canada, Europe, US), RCT Phase III | 507 | Overall: 45.2(NR)  10mg QW: 44.0(NR)  15mg QW: 45.3(NR)  Placebo: 46.5(NR) | Overall: 333(66%)  10mg QW: 120(60%)  15mg QW: 103(62%)  Placebo: 110(65%) | Dose reduction: IM alefacept 10mg QW for 12 weeks (n=173)  Standard treatment: IM alefacept 15mg QW for 12 weeks (n=166) | Through 24 weeks | **PASI 75 at week 14**  15mg: 21%  10mg: 12%  Placebo: 5%  (p<0.001 for 15mg vs. placebo and p=0.041 for 10mg vs. placebo) | ***Overall Response Rate (15mg, 10mg, placebo):**  **1) PASI 75**  **2) PASI 50**  **3) PGA “clear” or “almost clear”**  ***achieved at any time during treatment, retreatment or withdrawal periods**  1) 33%, 28%, 13%  (p<0.001 for 10mg and 15mg vs. placebo)  2) 57%, 53%, 35%  (p<0.001 for 15mg vs. placebo)  3) 24%, 22%, 8%  (p<0.001 for 10mg and 15mg vs. placebo) |
| Cafardi, et al., 2008 (US), Open-label [[19](#_ENREF_19)] | 16 | Cohort 1: 42.8(12.9)  Cohort 2: 43.6(11.2) | Cohort 1: 3(37.5%)  Cohort 2: 4(50%) | Dose escalation: Cohort 1 (n=8) received IM alefacept 30mg QW for 6 weeks followed by 15mg QW for 6 weeks and Cohort 2 (n=8) received IM alefacept 30mg QW for 12 weeks | Through 1 year after last treatment dose | **PASI 75 at week 14**  Cohort 1: 12.5% (1/8)  Cohort 2: 12.5% (1/8) | **PASI 50 at week 14**  Cohort 1: 3/8 (37.5%)  Cohort 2: 4/8 (50%) |
| **Withdrawal & Retreatment** | | | | | | | |
| Author, Year (Location) Study Design | N | Age mean (SD) | Gender n(%) male | Withdrawal Period | Retreatment Period | Primary Outcome | Secondary Outcome |
| Krueger et al., 2002 (Canada, US), RCT Phase III [[20](#_ENREF_20)] | 553 | Overall: 45(NR)  Cohort 1: 45(NR)  Cohort 2: 46(NR)  Cohort 3: 45(NR) | Overall: 387(70%)  Cohort 1: 131(72%)  Cohort 2: 129(70%)  Cohort 3: 127(68%) | 12 week withdrawal period after initial 12 weeks of alefacept  Cohort 2: After initial 12 weeks of drug treatment and following the 12 week withdrawal period, pts were permanently withdrawn from alefacept for the rest of study (n=184) | 12 weeks of retreatment after 12 weeks of withdrawal: IV alefacept 7.5mg QW^‖^  Cohort 1: Following the 12 week withdrawal period, pts were retreated with 12 weeks alefacept during the 12 week retreatment period (n=183)  Cohort 3: Pts received placebo for the initial 12 weeks and following the 12 week withdrawal period, pts were retreated with alefacept during the 12 week retreatment period (n=153) | **PASI-75 at week 2 of withdrawal period**  Initial 12 weeks treatment (cohorts 1 and 2): 14%  (p<0.001 vs. placebo)  Retreatment (cohort 1): 23%  (p<0.001 vs. placebo)  Withdrawal (cohort 2): 7%  Initial treatment and retreatment (cohort 1): 26% | **PASI 50 mean duration**  For pts who achieved PASI 75 in cohort 2, PASI 50 was maintained for a median duration of 216 days  PASI 50 median duration could not be calculated for cohort 1 because more than 50% of pts maintained PASI 50 at main endpoint (significant between cohorts 1 and 2 p=0.019)  ***Overall Response Rate:**  **1) PASI 75**  **2) PASI 50**  **3) PGA “clear” or “almost clear”**  ***achieved at any time during treatment, retreatment or withdrawal periods**  Initial 12 weeks treatment (cohorts 1 and 2):  1) 28%  2) 56%  3) 23%  Initial 12 weeks placebo (cohort 3):  1) 8%  2) 24%  3) 6%  p<0.001  Retreatment (cohort 1):  1) 37%  2) 64%  3) 30%  Withdrawal (cohort 2):  1) 19%  2) 49%  3) 18%  Initial treatment + Withdrawal (cohort 2):  1) 19%  2) 49%  3) 18%  Initial treatment + Retreatment (cohort 1):  1) 40%  2) 71%  3) 32% |
| Lowe et al., 2003 (US), Open-label [[21](#_ENREF_21)] | 174 | 45(NR) | NR(66%) | 12 week withdrawal period after 12 weeks of drug for up to 3 retreatment cycles | 12 weeks of retreatment after 12 weeks of withdrawal: IV alefacept 7.5mg QW  1^st^ cycle (n= 174)  2^nd^ cycle (n= 107)  3^rd^ cycle (n= 23) | **At week 2 of withdrawal period:**  **1) PASI 75**  **2) PASI 50**  **3) PGA “clear” or “almost clear”**  1^st^ cycle (n=170):  1) 16%,  2) 43%,  3) 11%  *1^st^ cycle, 2^nd^ cycle (n=50):  1) 16%, 18%  2) 44%, 52%  3) 10%, 14%  *Data for n=50 pts who completed both 1^st^ and 2^nd^ treatment cycles. | ***Overall Response Rate:**  **1) PASI 75**  **2) PASI 50**  **3) PGA “clear” or “almost clear”**  ***achieved at any time during treatment, retreatment or withdrawal periods**  1^st^ cycle (n=170):  1) 39%  2) 66%  3) 29%  *1^st^ cycle 🡪 2^nd^ cycle (n=50):  1) 32% 🡪 32%  2) 64% 🡪 68%  3) 20% 🡪 24% |
| Gordon et al., 2003 (Canada, Europe, US), Open-label [[22](#_ENREF_22)] | 131 | 46(NR) | 85(65%) | >12 weeks withdrawal period after 12 weeks of study drug in a Phase III trial (Ortonne/Lebwohl 2003) | 12 weeks of retreatment with IM alefacept 15mg after >12 weeks withdrawal from Phase III trial (Ortonne/ Lebwohl) when PGA of “mild” or worse (n=131) | **Median duration of PASI 50 response in PASI 75 responders of Phase III study (n=54)**  209 days (approximately 7 months)  **Median duration of PASI 50 response in PGA “clear” or “almost clear” responders of Phase III study (n=40)**  245 days (approximately 8 months) | **Overall Response Rate:**  **1) PASI 75**  **2) PASI 50**  **3) PGA “clear” or “almost clear”**  ***achieved at any time during retreatment or withdrawal periods**  1) 43%  2) 69%  3) 31% |
| Roberts et al., 2010 (Canada, Europe, US), Open-label [[23](#_ENREF_23)] | 183 | 1^st^ cycle: 45.8(12.6)  2^nd^ cycle: 45.6(12.7)  3^rd^ cycle: 45.3(11.9) | 1^st^ cycle: 116(66%)  2^nd^ cycle: 75(62%)  3^rd^ cycle: 53(60%) | 12 week withdrawal period after initial 12 weeks of drug and up to 2 retreatment courses | 12 weeks of retreatment after 12 weeks of withdrawal: IM alefacept 15mg QW  Up to 3 treatment and retreatment courses (1^st^, 2^nd^, 3^rd^ cycle) if disease was anything except “clear” on the PGA scale and CD4 T-cell counts were ≥ lower limit of normal  1^st^ cycle (n=175)  2^nd^ cycle (n=121)  3^rd^ cycle (n=88) | **Proportion of pts who achieved a PGA rating of “clear” or “almost clear” at week 2 and week 12 of withdrawal period:**  1^st^ cycle: 16%, 29%  2^nd^ cycle: 22%, 31%  3^rd^ cycle: 19%, 31% | **Proportion of pts who achieved a PGA rating of “clear” or “almost clear” at any time during a treatment cycle:**  1^st^ cycle: 35%  2^nd^ cycle: 42%  3^rd^ cycle: 42%  **Subanalysis:**  Pts “clear” or “almost clear” in 1^st^ cycle then same in:  2^nd^ cycle: 68%  3^rd^ cycle: 59%  Pts “clear” or “almost clear” in 2^nd^ cycle then same in:  3^rd^ cycle: 69%  Pts not “clear” or almost clear” in 1^st^ cycle then “clear” or “almost clear” in:  2^nd^ cycle: 27%  3^rd^ cycle: 32%  Pts not “clear” or almost clear” in 2^nd^ cycle then achieved “clear” or “almost clear” in:  3^rd^ cycle: 20%  **Median duration (days) of response (measured only for 1^st^ and 2^nd^ cycles) = period in days for which pts remained at a PGA response level without additional treatment**  “Clear” or “almost clear” at week 2 of withdrawal:  1^st^ cycle: 214  2^nd^ cycle: 126  “Mild or better” at week 2 of withdrawal:  1^st^ cycle: 340  2^nd^ cycle: 283  “Clear” or “almost clear” any time during treatment:  1^st^ cycle: 146  2^nd^ cycle: 123  “Mild or better” at any time during treatment:  1^st^ cycle: 287  2^nd^ cycle: 221  **Time to retreatment, duration (days) between last dose of one treatment cycle and the first dose of next treatment cycle, for pts who achieved ”clear” or “almost clear” at week 2 of withdrawal period**  1^st^ cycle:  Mean(SD): 281.9(155.24)  Median: 240.0  Range: 99-608  2^nd^ cycle:  Mean(SD): 218.8(133.39)  Median: 173.0  Range: 84-517 |

NR ^†^ = Not reported

QW ^††^ = Once weekly
