# Supplementary material for: Off-Label Biologic Regimens in Psoriasis: A Systematic Review of Efficacy and Safety of Dose Escalation, Reduction, and Interrupted Biologic Therapy
Source: PLoS One. 2012 Apr 11;7(4):e33486. doi: 10.1371/journal.pone.0033486 (PMC3324468; doi:10.1371/journal.pone.0033486)
Supplement: Table S6 — Safety Data for Etanercept Off-Label Regimens. (DOCX) [file pone.0033486.s006.docx]

| **Table S6. Etanercept: Safety Data for Off-Label Regimens** | | | | | | |
| --- | --- | --- | --- | --- | --- | --- |
| **Dose Escalation or Reduction** | | | | | | |
| Author, Year (Location) Study Design | Rebound/ Flares | Antidrug Antibodies | Serious Infection | Malignancy | Serious AE | Common AE |
| Leonardi et al., 2003 (US), RCT Phase III [[1](#_ENREF_1)] | NR^†^ | 8/652 (1.2%) pts tested positive for antibodies during the 24 week study (dose not specified) | NR | NR | NR | ISR, headache, URI, injection-site ecchymosis |
| Cassano et al., 2010 (Europe), Open-label [[2](#_ENREF_2)] | No reports of rebound or conversion of lesion morphology | NR | NR | NR | NR | NR |
| Leonardi et al., 2010, (Canada, Europe, US) Open-label [[3](#_ENREF_3)] | NR | 130/857 (15.2%) with serum samples had positive antibodies (dose not specified). All antibodies were non-neutralizing and developed to a similar degree in both groups of pts. 35/857 (3.8%) tested positive for antibodies 3+ times (dose not specified) | Serious Infections:  50mg QW^††^ only: 0.9 events/100 pt-years  50mg QW/BIW^¶^: 1.9 events/100 pt-years with 2.0 in QW and 1.8 in BIW exposure  17 infections in 12/912 pts:  50 QW: 3 pneumonia  50 QW/BIW: 2 cellulitis  50 QW/BIW: 12 cases  QW exposure = appendiceal abscess, appendicitis, gastroenteritis and influenza, gastritis, pain in extremity and skin discoloration  BIW exposure = streptococcal fasciitis + myositis + septic shock, diverticulitis, hepatitis C | Rate of malignancy: 1.5 events/100 pt-years (dose not specified)  6 Malignancies  2 malignant neoplasms, 2 metastatic neoplasms of unknown origin, bladder cancer, prostate cancer (dose not specified) | 59 Serious AE in 46/912 pts:  50mg QW only: 2 cases (2/321) subdural hematoma  50mg QW/BIW: 2 cases MI (2/591), 2 cases (2/591) nephrolithiasis  1 death: metastatic neoplasm of unknown origin (dose not specified) | Labs: 19 results with grade-3 or -4; 14 of these results were liver enzyme abnormalities |
| **Withdrawal & Retreatment** | | | | | | |
| Author, Year (Location) Study Design | Rebound/ Flares | Anti-drug Antibodies | Serious Infection | Malignancy | Other Serious AE | Common AE |
| Gordon et al., 2006 (US), RCT [[4](#_ENREF_4)] | 1 case (1/85) of rebound during study drug discontinuation period in the 25mg QW group, reversed after 4 weeks of retreatment | 12/297 (4.7%) pts had antibodies during retreatment phase (dose not specified)  All were low titers and all were non-neutralizing | 2 Serious Infections 50mg BIW (2/103):  cellulitis, osteomyelitis | NR | 7 Serious AE:  All in retreatment period (dose not specified) | URI, flu syndrome, headache, hypertension, sinusitis, anxiety, gastroenteritis  ISR: 67/486(14%) by week 12 of initial treatment with any dose of etanercept  15/342(4%) in retreatment with any dose of etanercept  Labs: three grade-3 abnormalities (2 AST, 1 AST and ALT) |
| Moore et al., 2007 (US), EASE RCT & open-label [[5](#_ENREF_5)] | NR | NR | NR | 5 malignancies 5/2456 (0.2%): prostate (1 continuous, 1 interrupted), adenocarcinoma (1 interrupted), glioblastoma multiforme (1 interrupted), breast cancer (1 continuous)  16 Non-melanoma skin cancer: squamous cell carcinoma (5 continuous, 4 interrupted), basal cell carcinoma (2 continuous, 5 interrupted) | 79 Serious AE  79/2546 (3.1%): 6 Non-cardiac chest pain (3 each continuous and interrupted), 4 dyspnea (all continuous), 3 depression, 3 CAD, 3 uterine leiomyoma, (other serious AE not specified)  3 CHF (dosing interval not specified)  1 death: pneumonia, immunosuppression and sepsis was possibly related to study drug during the initial 12 weeks of treatment | Common AE:  Continuous: 688 (54.1%)  Interrupted: 671 (52.7%)  Injection site erythema, headache arthralgia, nasopharyngitis |
| Ortonne et al., 2009 (NR), CRYSTEL RCT & open-label *post hoc analysis* [[6](#_ENREF_6)] | NR | NR | 1 case (1/363) in the paused treatment arm | NR | Serious AE:  8.5% (31/363) in the paused treatment arm | Paused treatment arm: URI (19%), headache (14%) most common |

NR ^†^ = Not reported

QW ^††^ = Once weekly

BIW ^¶^ = Twice weekly

ISR = Injection site reaction; URI = Upper respiratory infection; AST = aspartate aminotransferase; ALT = alanine transaminase; CHF = Congestive heart failure
