# Supplementary material for: Off-Label Biologic Regimens in Psoriasis: A Systematic Review of Efficacy and Safety of Dose Escalation, Reduction, and Interrupted Biologic Therapy
Source: PLoS One. 2012 Apr 11;7(4):e33486. doi: 10.1371/journal.pone.0033486 (PMC3324468; doi:10.1371/journal.pone.0033486)
Supplement: Table S7 — Safety Data for Adalimumab Off-Label Regimens. (DOCX) [file pone.0033486.s007.docx]

| **Table S7. Adalimumab: Safety Data for Off-Label Regimens** | | | | | | |
| --- | --- | --- | --- | --- | --- | --- |
| **Dose Escalation** | | | | | | |
| Author, Year (Location) Study Design | Rebound/ Flares | Anti-drug Antibodies | Serious Infection | Malignancy | Other Serious AE | Common AE |
| Gordon et al., 2006 (Canada, US) RCT [[7](#_ENREF_7)] | NR^†^ | NR | 2 Serious Infections:  QW^††^ (1/50): recent-onset latent TB infection  EOW^§^ (1/97): coccidioidomycosis | 5 Malignancies:  QW (3/50)**:** breast carcinoma, gastric adenocarcinoma, malignant melanoma  EOW (2/97): malignant melanoma, squamous cell carcinoma | 14 Serious AE:  QW (8/50): migraines, bronchitis, osteoarthritis, kidney stones, palpitations, coronary artery disease, 2 cerebrovascular accidents  EOW (3/97): accidental fall, malignant melanoma, squamous cell carcinoma  1 death (1/50) from cerebrovascular accident in weekly adalimumab | Labs: 2 pts discontinued because liver function enzymes elevated 3-3.5 times the upper limit of normal (adalimumab, dose not specified). 1 pt discontinued because hematocrit was moderately decreased |
| **Withdrawal & Retreatment** | | | | | | |
| Author, Year (Location) Study Design | Rebound/ Flares | Anti-drug Antibodies | Serious Infection | Malignancy | Other Serious AE | Common AE |
| Menter et al., 2008 (Canada, US), RCT Phase III [[8](#_ENREF_8)] | No reports of rebound after treatment withdrawal | 73/825 (8.8%) of pts in the all adalimumab group* had detectable AAAs at least once during 52-week trial. 3/7 AAA-positive pts compared with 65/233 (28%) of AAA-negative pts lost an adequate response. Antibodies were not correlated with any treatment related AEs, including SAEs, and ISRs.  *All adalimumab group = all pts who received at least one dose of adalimumab during the 52-week trial | Soft tissue infections  All adalimumab group 12/825 (1.5%): Cellulitis, abscess, TB (1 case), oral candidiasis (1 case), other infections not specified | 7 Non-melanoma skin cancers  All adalimumab group (7/825): basal cell carcinomas (3), squamous cell carcinomas (3), atypical endophytic epidermoid proliferation (1) | NR | NR |
| Papp et al., 2011 (Canada, Europe, US), Open-label [[9](#_ENREF_9)] | No reports of rebound after treatment withdrawal | Prior to withdrawal of treatment 0/272 (0%) of mITT-R pts had AAAs.  After withdrawal and prior to retreatment 17/275 (6%) had AAAs.  After retreatment, AAA-positive samples decreased to 3/264 (1%) at week 12 of retreatment, and 4/262 (2%) at week 16 of retreatment.  3 pts not AAA-positive at week 0 of retreatment were AAA-positive at week 12 of retreatment (1) and week 16 of retreatment (2)  Among pts who relapsed, those who achieved PGA of 0/1 at week 16 of retreatment 5/119 (4%) were AAA-positive compared to those who did not achieve PGA 0 or 1, 8/55 (15%)  AAAs was not reported to be associated with increased risk of hypersensitivity reactions | 2 Serious Infections 2/285 (0.7%) during the retreatment period: Pneumonia, hepatitis C | 1 Malignancy  1/285 (0.4%) during the retreatment period: malignant melanoma in situ | 7 Serious AE  7/285 (2.5%) during the retreatment period: coronary artery disease, tendon rupture, malignant melanoma in situ, abdominal adhesions, umbilical hernia, chest discomfort, nephrolithiasis, fracture of humerus | 1 ISR  1/285 (0.4%) during the retreatment period in an AAA-negative pt |

NR ^†^ = Not reported

QW ^††^ = Once weekly

EOW ^§^ = Every other week

AAAs = Anti-adalimumab antibodies; ISR = Injection site reaction
