# Supplementary material for: Off-Label Biologic Regimens in Psoriasis: A Systematic Review of Efficacy and Safety of Dose Escalation, Reduction, and Interrupted Biologic Therapy
Source: PLoS One. 2012 Apr 11;7(4):e33486. doi: 10.1371/journal.pone.0033486 (PMC3324468; doi:10.1371/journal.pone.0033486)
Supplement: Table S8 — Safety Data for Infliximab Off-Label Regimens. (DOCX) [file pone.0033486.s008.docx]

| **Table S8. Infliximab: Safety Data for Off-Label Regimens** | | | | | | |
| --- | --- | --- | --- | --- | --- | --- |
| **Dose Escalation** | | | | | | |
| Author, Year (Location) Study Design | Rebound/ Flares | Antidrug Antibodies | Serious Infection | Malignancy | Serious AE | Common AE |
| Chaudhari et al., 2001 (US), RCT [[10](#_ENREF_10)] | NR^†^ | NR | 2 Infections  5mg/kg (1/11): Dental abscess  10mg/kg (1/11): Pneumonia | NR | NR | Headache, URI, abdominal bloating or pain  Labs: elevated AST 10mg/kg (2/11) vs. placebo (1/11)  Antinuclear Antibodies: 5mg/kg (2/11): titers of 1:160 and 1:320 |
| **Withdrawal & Retreatment** | | | | | | |
| Author, Year (Location) Study Design | Rebound/ Flares | Antidrug Antibodies | Serious Infection | Malignancy | Serious AE | Common AE |
| Gottlieb et al., 2003 (US), Open-label [[11](#_ENREF_11)] | NR | NR | 7 Serious Infections:  10mg/kg (3/11)  5mg/kg (2/11)  Placebo (2/11)  2 cases cellulitis, tooth abscess, ear infection, infected wisdom tooth, bronchitis, pneumonia (infection not specified to treatment group or treatment period) | NR | 2 Severe Infusion reactions:  5mg/kg (2/11) (treatment interval not specified) | Infusion reaction  3/33 (9%) retreatment period (dose not specified) |
| Gottlieb et al., 2004 (US), RCT [[12](#_ENREF_12)] | NR | 3mg/kg: 21/76 (27.6%)  5mg/kg: 17/87 (19.5%) had antibodies to infliximab  9/38 (24%) with antibodies experienced infusion reactions through week 26 compared to 25/116 (22%) with negative antibodies | 1 Serious Infection:  5mg/kg (1/99): Sepsis | 1 Malignancy:  3mg/kg (1/98): Squamous cell carcinoma | 3 Pts with Serious AE  3mg/kg (1/98): cholecystitis and cholelithiasis  5mg/kg (2/99): diverticulitis; sepsis, and pyelonephritis  2 Severe Infusion reactions:  5mg/kg (2/99) | Labs: elevated ALT infliximab (34%) vs. placebo (16%); elevated AST infliximab (24%) vs. placebo (14%)  Anti-dsDNA antibodies: Infliximab: 7/197 (3.8%)  Placebo: 1/51 (2.1%) |
| Menter et al., 2007 (Canada, Europe, US), EXPRESS II RCT [[13](#_ENREF_13)] | NR | 3m/kg every-8-week: 69/145 (51.5%)  3mg/kg interrupted: 60/147 (46.2%)  5mg/kg every-8-week: 49/148 (35.8%)  5mg/kg interrupted: 59/149 (41.5%)  Majority of titers (61.1%) were <1:40  In all retreatment groups (every-8-week and interrupted treatment groups) PASI responders at week 10 were less likely to maintain response at week 50 if positive antibodies. Positive antibodies had a greater risk of infusion reaction (2/5) than absence of antibodies (0/5). | 2 Serious Infections  3mg/kg induction phase (1/313): 1 case TB  5mg/kg interrupted treatment group (1/149): 1 case TB | 12 Malignancies:  1 case each of breast carcinoma, salpingeal adenocarcinoma, squamous cell carcinoma; 9 cases of basal cell carcinomas (dose not specified) | 3 Lupus-like syndrome:  Infliximab: 2 cases (dose not specified)  Placebo: 1 case  1 Peripheral Neuropathy considered possibly related to study drug (dose not specified)  9 possible delayed hypersensitivity reactions, 4 serious (dose not specified)  Infusion reactions: 5 serious reactions (dose not specified) | URI, headache, pharyngitis, rhinitis, sinusitis, cough  Labs:  Elevated ALT 4.9%, AST 3.1% (dose not specified). ALT elevated in 4.1% in all infliximab retreatment groups except 7.4% in 5mg interrupted |

NR ^†^ = Not reported

URI = Upper respiratory infection; AST = aspartate aminotransferase; ALT = alanine transaminase
