# Supplementary material for: Off-Label Biologic Regimens in Psoriasis: A Systematic Review of Efficacy and Safety of Dose Escalation, Reduction, and Interrupted Biologic Therapy
Source: PLoS One. 2012 Apr 11;7(4):e33486. doi: 10.1371/journal.pone.0033486 (PMC3324468; doi:10.1371/journal.pone.0033486)
Supplement: Table S9 — Safety Data for Ustekinumab Off-Label Regimens. (DOCX) [file pone.0033486.s009.docx]

| **Table 9. Ustekinumab: Safety Data for Off-Label Regimens** | | | | | | |
| --- | --- | --- | --- | --- | --- | --- |
| **Dose Escalation** | | | | | | |
| Author, Year (Location) Study Design | Rebound/ Flares | Antidrug Antibodies | Serious Infection | Malignancy | Serious AE | Common AE |
| Papp et al., 2008 (Europe, North America), RCT Phase III [[14](#_ENREF_14)] | NR^†^ | 20/158 (12.7%) partial responders had antibodies to drug compared with 12/589 (2.0%) PASI 75 responders at week 52 of the study. Overall, 65/1202 (5.4%) developed antibodies. Most antibodies were neutralizing and no cases were associated with ISRs. | 1 Serious Infection 1/81 (1.2%) in every-12-week ustekinumab in dose intensification phase | 2 Malignancies  2/77 (2.6%) in every-8-week ustekinumab in dose intensification phase: 1 non-cutaneous, 1 cutaneous | 8 Serious AEs in dose intensification phase  2/77 (2.6%) every-8-weeks  6/81 (7.4%) every-12-weeks | Nasopharyngitis, URI, injection site erythema, arthralgia, cough |
| **Withdrawal & Retreatment** | | | | | | |
| Author, Year (Location) Study Design | Rebound/ Flares | Antidrug Antibodies | Serious Infection | Malignancy | Serious AE | Common AE |
| Leonardi et al., 2008 (Belgium, Canada, US), RCT Phase III [[15](#_ENREF_15)] | No reports of rebound after treatment withdrawal | 38/746 (5.1%) had antibodies at evaluation at week 76 (dose and dose interval not specified). Antibodies were predominantly low titer (<1/320) and not associated with any ISRs | 2 Serious infections 2/160 (1.2%) in interrupted ustekinumab: gastroenteritis (other infection not specified) | 1/160 (0.6%) Non-cutaneous cancer- colon cancer- in the interrupted treatment group  2/161 (1.2%) Cutaneous Malignancies in the continuous treatment group (not specified) | 8 Serious AE:  7/160 (4.4%) in the interrupted treatment group  1/160 (0.6%) in the continuous treatment group | URI, nasopharyngitis, arthralgia, headache |

NR ^†^ = Not reported

URI = Upper respiratory infection; ISR = Injection site reaction
