# Supplementary material for: Off-Label Biologic Regimens in Psoriasis: A Systematic Review of Efficacy and Safety of Dose Escalation, Reduction, and Interrupted Biologic Therapy
Source: PLoS One. 2012 Apr 11;7(4):e33486. doi: 10.1371/journal.pone.0033486 (PMC3324468; doi:10.1371/journal.pone.0033486)
Supplement: Table S10 — Safety Data for Alefacept Off-Label Regimens. (DOCX) [file pone.0033486.s010.docx]

| **Table S10. Alefacept: Safety Data for Off-Label Regimens** | | | | | | |
| --- | --- | --- | --- | --- | --- | --- |
| **Dose Escalation or Reduction** | | | | | | |
| Author, Year (Location) Study Design | Rebound/ Flares | Antidrug Antibodies | Serious Infection | Malignancy | Serious AE | Common AE |
| Gribetz et al., 2005 (US), RCT & open-label [[16](#_ENREF_16)] | No reports of rebound after treatment withdrawal | NR^†^ | 2 Infections:  Cohort 1 (2/10): cellulitis, *Helicobacter pylori* | NR | NR | Cohort 1 (2/10): 2 cases URI  Cohort 2 (7/10): 6 cases URI in 4 pts, 1 case bronchitis |
| Lebwohl et al., 2003 [[17](#_ENREF_17)] and Ortonne et al., 2003 [[18](#_ENREF_18)] (Canada, Europe, US), RCT Phase III | No reports of rebound during 12-week treatment withdrawal | 10/171 (6%) in the 10mg group and 4/161 (2%) in 15mg group had antibodies after the last dose of alefacept. Titers were low (<1:40), non-neutralizing, and were not associated with hypersensitivity reactions | Infections:  15mg: 26/166 (16%)  10mg: 25/173 (14%)  Placebo: 19/168 (11%)  Alefacept infections included: viral infection, flu-like syndrome, pharyngitis, sinusitis, bronchitis, bacterial infection, HSV (dose not specified)  No correlation between CD4 counts and infection | 3 Malignancies  15mg (2/166): 2 cases basal cell carcinoma  Placebo (1/168): prostatic carcinoma | Serious AE  15mg: 7/166 (4%)  10mg: 8/173 (5%)  Placebo: 10/168 (6%) | Headache, pruritus, pharyngitis, accidental injury  ISR: 7% of those treated with alefacept |
| Cafardi, et al., 2008 (US), Open-label [[19](#_ENREF_19)] | 2 cases (2/8) of morphological change from plaque psoriasis to erythroderma in cohort 1. One case during 30mg phase, one case during 15mg phase | NR | Infections:  Cohort 1: 6/8  Cohort 2: 4/8  Infections ranged from URI to pneumonia (other infections not specified, dose for each infection not specified) | NR | 2 Serious AE:  Cohort 1 (1/8): erythroderma and severe clinical presentation during 15mg alefacept phase necessitated hospitalization  (1/16) chest pain (dose not specified)  CD4+ cell count <250 cells/mm:  Cohort 1: 2/8 | Headache, pruritus, erythroderma, dizziness, nausea |
| **Withdrawal & Retreatment** | | | | | | |
| Author, Year (Location) Study Design | Rebound/ Flares | Antidrug Antibodies | Serious Infection | Malignancy | Serious AE | Common AE |
| Krueger et al., 2002 (Canada, US), RCT Phase III [[20](#_ENREF_20)] | No reports of rebound after treatment withdrawal | Anti-alefacept antibodies in <1% of pts in each cohort. Titers were low (<1:40) and there were no safety concerns about immune hypersensitivity | No infections associated with low CD4+ T-cell counts (<250) | NR | NR | Accidental injury, headache, pharyngitis, rhinitis, pruritus  Labs: elevated ALT in cohort 1: 21/122(17%) and cohort 2: 9/114(8%)  0-12 weeks:  cohorts 1 and 2 had higher incidence of chills vs. placebo (10% vs. 1%) |
| Lowe et al., 2003 (US), Open-label [[21](#_ENREF_21)] | No reports of rebound after treatment withdrawal. No flares of disease | 3/174 (1.7%) in the 1^st^ cycle and 1/107 (0.9%) in the 2^nd^ cycle tested positive for anti-alefacept antibodies. Low titers were reported in all cases. | 2 Serious Infections:  1^st^ cycle: 1/174 (0.6%), pneumonia  2^nd^ cycle: 1/107 (0.9%), Herpes zoster | 5 Malignancies:  1 adenocarcinoma of colon, 1 adenocarcinoma of lung, 3 squamous cell carcinoma of skin (dose cycle not specified) | 3 Serious AE:  1^st^ cycle: 2/174 (1.1%), 2 cases low CD4+ T-cell counts  2^nd^ cycle: 1/107 (0.9%), pulmonary fibrosis | Pharyngitis, rhinitis, accidental injury, viral infection |
| Gordon et al., 2003 (Canada, Europe, US), Open-label [[22](#_ENREF_22)] | NR | 3/127 (2%) tested positive for anti-alefacept antibodies during retreatment. Low titers were reported in all cases. | NR | NR | NR | Accidental injury, pharyngitis, rhinitis, headache |
| Roberts et al., 2010 (Canada, Europe, US), Open-label [[23](#_ENREF_23)] | NR | 2/175 (1.1%) tested positive for anti-alefacept antibodies at any time during the study. Both had low titers.  1/175 (0.6%) in 1^st^ cycle did not continue in 2^nd^ or 3^rd^ cycles. 1/175 (0.6%) tested positive for antibodies in all 3 cycles. | 15 Serious Infections:  1 case mycoplasmal tracheo-bronchitis, 14 cases herpes simplex virus: 1^st^ cycle: 4/175 (2%), 2^nd^ cycle: 6/121 (5%), 3^rd^ cycle: 4/88 (5%) | 7 Malignancies  1 case Non-Hodgkin lymphoma, 1 case Hodgkin disease, others not specified (cycle not specified)  9 Non-melanoma skin cancers in 5 pts: 3 squamous cell, 6 basal cell carcinomas (cycle not specified) | Serious AE:  1^st^ cycle: 5%  2^nd^ cycle: 3%  3^rd^ cycle: 0%  Atrial fibrillation, cholelithiasis, hemoptysis, Hodgkin disease, leukopenia, lymphadenopathy, lymphoma, mycoplasmal tracheo-bronchitis, MI, neutropenia, pancreatitis | Nasopharyngitis influenza, URI, arthralgia, headache |

NR ^†^ = Not reported

URI = upper respiratory infection; ISR = injection site reaction; ALT = alanine transaminase
